# mRNA markers for survival prediction in glioblastoma multiforme patients: a systematic review with bioinformatic analyses

**DOI:** 10.1186/s12885-024-12345-z

**Published:** 2024-05-21

**Authors:** Parisa Azimi, Taravat Yazdanian, Abolhassan Ahmadiani

**Affiliations:** 1 Neurosurgeon, Neuroscience Research Center, Shahid Beheshti University of Medical Sciences, Arabi Ave, Daneshjoo Blvd, Velenjak, Tehran 19839- 63113 Iran; 2 School of Medicine, Capital Medical University, Beijing, China

**Keywords:** Glioblastoma, mRNA, Systematic review, Overall survival, Bioinformatics

## Abstract

**Background:**

Glioblastoma multiforme (GBM) is a type of fast-growing brain glioma associated with a very poor prognosis. This study aims to identify key genes whose expression is associated with the overall survival (OS) in patients with GBM.

**Methods:**

A systematic review was performed using PubMed, Scopus, Cochrane, and Web of Science up to Journey 2024. Two researchers independently extracted the data and assessed the study quality according to the New Castle Ottawa scale (NOS). The genes whose expression was found to be associated with survival were identified and considered in a subsequent bioinformatic study. The products of these genes were also analyzed considering protein-protein interaction (PPI) relationship analysis using STRING. Additionally, the most important genes associated with GBM patients’ survival were also identified using the Cytoscape 3.9.0 software. For final validation, GEPIA and CGGA (mRNAseq_325 and mRNAseq_693) databases were used to conduct OS analyses. Gene set enrichment analysis was performed with GO Biological Process 2023.

**Results:**

From an initial search of 4104 articles, 255 studies were included from 24 countries. Studies described 613 unique genes whose mRNAs were significantly associated with OS in GBM patients, of which 107 were described in 2 or more studies. Based on the NOS, 131 studies were of high quality, while 124 were considered as low-quality studies. According to the PPI network, 31 key target genes were identified. Pathway analysis revealed five hub genes (IL6, NOTCH1, TGFB1, EGFR, and KDR). However, in the validation study, only, the FN1 gene was significant in three cohorts.

**Conclusion:**

We successfully identified the most important 31 genes whose products may be considered as potential prognosis biomarkers as well as candidate target genes for innovative therapy of GBM tumors.

**Supplementary Information:**

The online version contains supplementary material available at 10.1186/s12885-024-12345-z.

## Introduction

Glioblastoma multiforme (GBM) is one of the most malignant gliomas of the central nervous system (CNS) [1]. The disappointing outcome of GBM treatment is a median survival of only 15 months despite multi-modalities of treatments [2, 3]. Based on the literature, GBM has special biological characteristics presenting high heterogeneity, diffusing invasiveness, and capacity to resist conventional therapies. In addition, the existence of biological barriers, e.g., BBB, makes this tumor difficult to treat [4]. Hence, the development of new methods for the clinical treatment of GBM may be facilitated by identifying the key genes associated with GBM prognosis [5].

Over the last decade, an increased focus has been on clarifying the origin, genomic landscape, and gene expression profile of GBM by identifying specific molecular markers and pathways involved in this pathology [6]. The advent of large-scale transcriptomic analyses in various cancers has tremendously increased our understanding of tumor biology and possible cancer therapy approaches [4]. Accordingly, in recent years, an increasing number of studies have focused on gene expression patterns to propose biomarkers and GBM tumor treatment strategies [7]. However, most of this information has not been translated into clinical practice for GBM patients [7].

The vast quantities of genomic data are now being deposited in public database repositories, such as Array Express (https://www.ebi.ac.uk/arrayexpress/), The Cancer Genome Atlas (TCGA, https://portal.gdc.cancer.gov), Chinese Glioma Genome Atlas (CGGA, http://www.cgga.org.cn) and Gene Expression Omnibus (GEO, https://www.ncbi.nlm.nih.gov/geo/). These genomic data are used by researchers around the world for the discovery of new genes of interest in GBM tumors. Several studies considered numerous mRNA expression datasets and identified gene signature panels to estimate prognosis in GBM tumors to improve the prognostic and predictive assessment of the tumors [8–262]. However, there is no consensus in the literature on the top gene sets that could be eventually used in clinical practice.

Considering the current state of our knowledge, we sought that a systematic survey of the literature is urgently required to identify genes whose expression could be predictive of GBM survival. Subsequently, to determine the top genes whose expression could be of interest in clinical practice, we assess biological pathways and protein-protein interaction (PPI) networks associated with these genes via bioinformatic analyses.

## Materials and methods

The Preferred Reporting Items for Systematic Reviews and Meta-Analysis (PRISMA) guideline [263] was followed to conduct the review (Supplementary File 1, Table S1.). PubMed, Scopus, Cochrane, and Web of Science databases were used to search for relevant studies published between 24th February 2003 and 1st January 2024. The search was conducted by the terms “gene expression” or “expressed genes” or “mRNA” or “RNA-Seq” and “survival” or “prognostic” or “biomarker” and “Glioblastoma multiforme (GBM)” or “high-grade glioma”. The full search strategy is reported in the supplementary File 1, Table S2.

Inclusion criteria were: (a) clinical study with human participants, (b) bioinformatics analysis study, (c) full-text articles, (d) published in the English language, (e) published in peer-reviewed journals, and (f) only genes related to GBM were considered. The exclusion criteria were as follows: (a) reviews, letters to the editor, and abstracts, (b) duplicate publications, (c) Plasma biomarker study, (d) participants with immunohistochemical (IHC) and Western blot analysis, (e) cell line study, (f) studies that did not observe a significant correlation between mRNA expression and overall survival, (g) recurrent glioblastoma, (h) pseudogene, and (i) animal study and progression-free survival (PFS) were not considered.

### Data extraction and quality assessment

Two independent authors (PA and TA) assessed and extracted all relevant articles. For each study, the following items were extracted: first author, publication year, country, mRNA, increased expression, decreased expression, public gene databases, detection method, short survival, long survival, and area under curve (AUC) for gene panel. The Newcastle–Ottawa Scale (NOS) was used to evaluate the quality of the eligible articles for case-control studies. NOS involves three perspectives: study group selection, group comparability, and whether the exposure or the outcome of interest for a case–control study is listed in the scale. Each study can obtain a maximum of nine stars [264]. Studies scoring above the median NOS value were considered as high quality (low risk of bias) and those scoring below the median value were considered as low quality (high risk of bias). A summary of the method of quality evaluation is presented in Table 1.

**Table 1:** Check list for quality evaluation and scoring of studies based on NOS

Check list
*Selection* 1. Is the case definition adequate? (if yes, one star) 2. Representativeness of the sample. Truly representative or somewhat representative? (if yes, one star) 3. How representative was the bioinformatic analysis group in comparison with the validation group, and were assess by mRNA expression? (if yes, one star; no star if the patients were selected only in one group) 4. Use of bioinformatic database analysis and specimen verification as RT-qPCR to identify novel biomarkers predicting survival in GBM tumors. Are both of them used? (if yes, one star)
*Comparability* Comparability of bioinformatic analysis dataset results with other datasets or methods of measurements as RT-qPCR basis of the design or analysis (if yes, two stars; one star was assigned if validation and verification was not reported clearly)
*Outcome assessment* 6. Ascertainment of the outcome: clearly defined outcome of mRNA expression, survival analysis, and methods of measurements as RT-qPCR, (yes, two stars for information ascertained; one star if two of this information were not reported) 7. Appropriate statistical analysis: The statistical test used to analyze mRNA and the survival of GBM patients as bioinformatic analysis, RT-qPCR, or microarray were clearly defined and appropriate for bioinformatic analysis group or verification group (if yes, one star; no star was assigned if outcomes were not reported)

mRNA, messenger RNA; RT-qPCR, Reverse transcription-quantitative polymerase chain reaction

### Bioinformatic and statistical analysis

#### Protein–protein interaction (PPI) network and signaling pathways analysis

All 613 genes (with *p* < 0.05) obtained from this review study were considered in the bioinformatic analysis. The PPI network was constructed by Cytoscape software (version 3.9.0; https://cytoscape.org/). The top important nodes of the PPI network were obtained based on the Cytohubba plug-in. The 5 well-known central indices, including degree, stress, betweenness, closeness, and radiality of nodes, were considered to rank the network nodes. The top 10% of genes were determined in each metric. Then, common genes were identified between five metrics. Finally, between common genes, proteins with a high degree of centrality were selected and were considered the most important ones to investigate their association with survival in GBM patients. Moreover, the top 10 genes ranked by degree are calculated.

A pathway analysis using the GO Biological Process (GOBP) 2023 database through the ENRICHR package (https://maayanlab.cloud/Enrichr/, accessed on 23 March 2024) was then performed for further specified related mechanisms involved in cancer such as cell proliferation, differentiation, apoptosis, mitosis, angiogenesis, and stemness. Only GOBP terms with adjusted p-value < 0.01 by ENRICHR analysis were used.

### Survival analysis and validation of the gene expression in the GEPIA2 and CGGA datasets

To confirm the reliability of the identified gene from the PPI network, Kaplan-Meier curves were created according to the GEPIA2 (http://gepia2.cancer-pku.cn) and the CGGA (http://www.cgga.org.cn) databases. CGGA contained two glioma data sets, namely, mRNAseq_325 and mRNAseq_693. Primary GBM of CGGA (mRNAseq_325) and CGGA (mRNAseq_693) data were considered. To determine differences in overall survival for patients with a low and high gene-expressing GBM, OS Kaplan-Meier analysis was performed by the GEPIA2 using the TCGA gene expression dataset and CGGA online applications. Kaplan–Meier curves were generated with a 50% median expression cutoff for high- and low-expressing groups. The estimation of hazard ratios was done by Cox proportional hazards model regression analysis. A 95% confidence interval was set and used. *P* < 0.05 was a statistically significant difference in validation cohorts from GEPIA2 and CGGA.

## Results

### Descriptive statistics

**Fig. 1:**
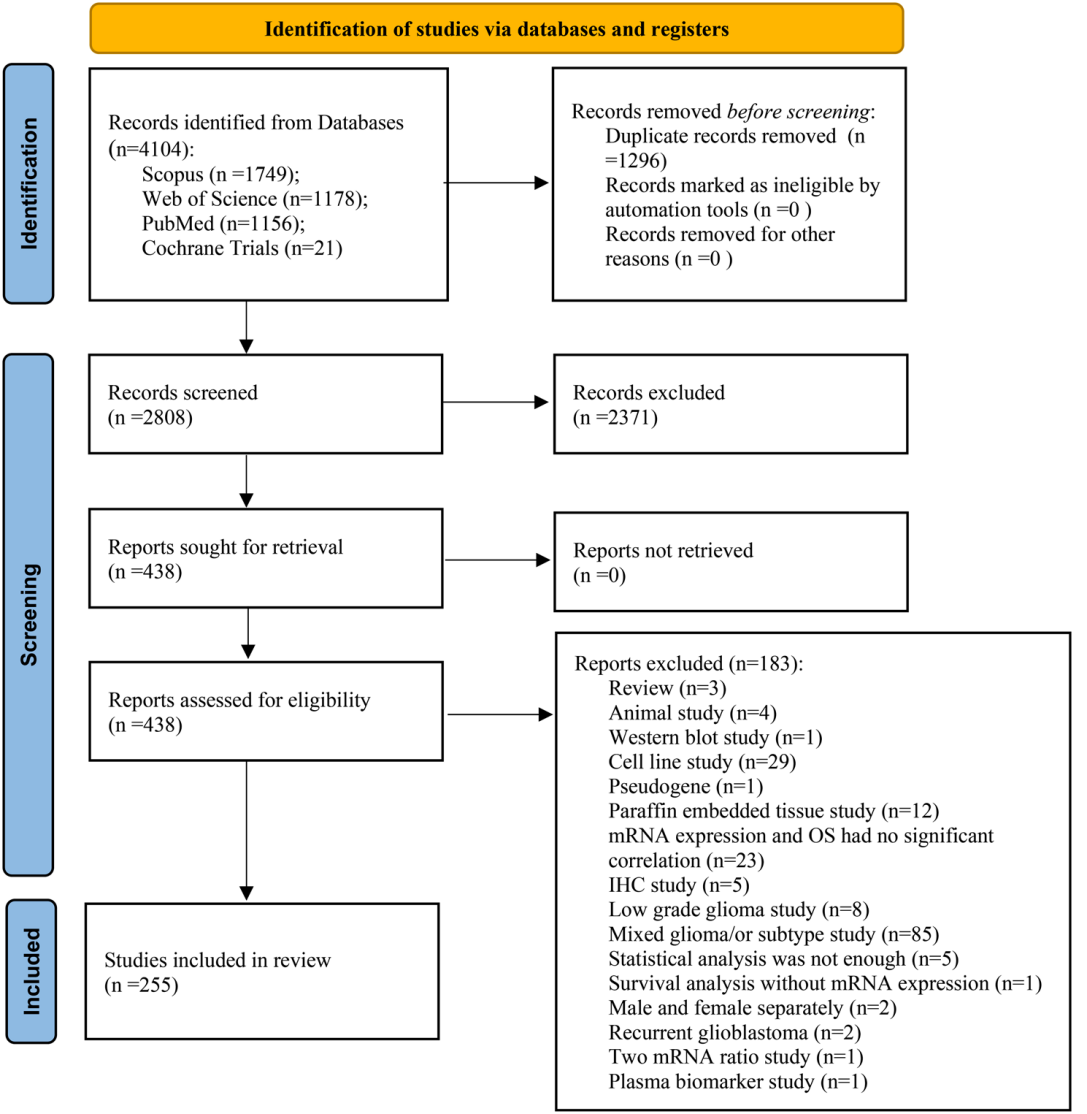
Flowchart of the selection process

The workflow of the literature selection process is shown in Fig. 1. In brief, 4104 articles were found via an initial literature search of the databases, and 1296 studies were excluded owing to duplication. After screening the titles and abstracts, 2371 studies were not considered relevant to the purpose of this systematic review based on method. Subsequently, 255 studies were enrolled, the characteristics of each study were shown in Supplementary Table 2. Among these, 161 studies were conducted in China [16, 18, 20–21, 23–29, 31, 34, 36–37, 40–44, 47–48, 50, 53, 55, 64, 66–69, 71–74, 80, 82–87, 89, 92–95, 97–98, 102–105, 107, 109–113, 115–118, 120–124, 126–127, 135–137, 139–144, 146–152, 154–155, 157, 160–164, 166, 168–170, 172–173, 177–179, 184–190, 193–195, 197–197, 206–208, 212–213, 215–221, 223–226, 229, 231–246, 244, 246–257, 259–261 ], 23 studies were conducted in USA [10, 13–14, 32, 45–46, 58, 63, 75, 77, 88, 106, 108, 114, 133–134, 159, 165, 182, 192, 228, 243, 245]; besides that, India [9, 19, 30, 33, 59, 101, 130, 132, 196, 205], Taiwan [61, 79, 96, 145, 156, 175–176, 242], Germany [38, 51, 65, 90, 180, 230], Japan [22, 57, 76, 129, 258], Republic of Korea [62, 167, 171, 209, 241], UK [39, 60, 131, 191], Spain [15, 119, 153, 227], Lithuania [49, 99, 125, 138], Italy [100, 183, 203], France [ 8,12,158], Slovenia [11, 81, 128], Switzerland [35, 52], Sweden [78, 174], Turkey [211, 214], Russia [222], Finland [17], Netherland [204], Hungary [54], Canada [70], Brazil [210] Iran [262], and Austria [181]. Among 255 studies, 192, and 37 of them used the dataset of the TCGA and the CGGA, respectively.

In the 720 genes studies, 613 unique genes were identified whose expression was associated with overall survival in GBM, of which 107 were described in two or more studies. See Supplementary Table 2 for details about the number of studies that described each gene, and whether or not it was found to be upregulated, downregulated, and the databases used.

### NOS assessment

The risk of bias evaluation of the included studies for case-control studies according to the NOS is shown in Supplementary Tables 2 and Supplementary File 1, Table S4. Based on the NOS, the median score of the included studies was 7. Among the 255 studies, 131 studies that scored ≥ 7 were considered to present a low risk of bias. 124 of the studies were considered with a high risk of bias since they scored b < 7.

### Bioinformatic analysis

**Fig. 2:**
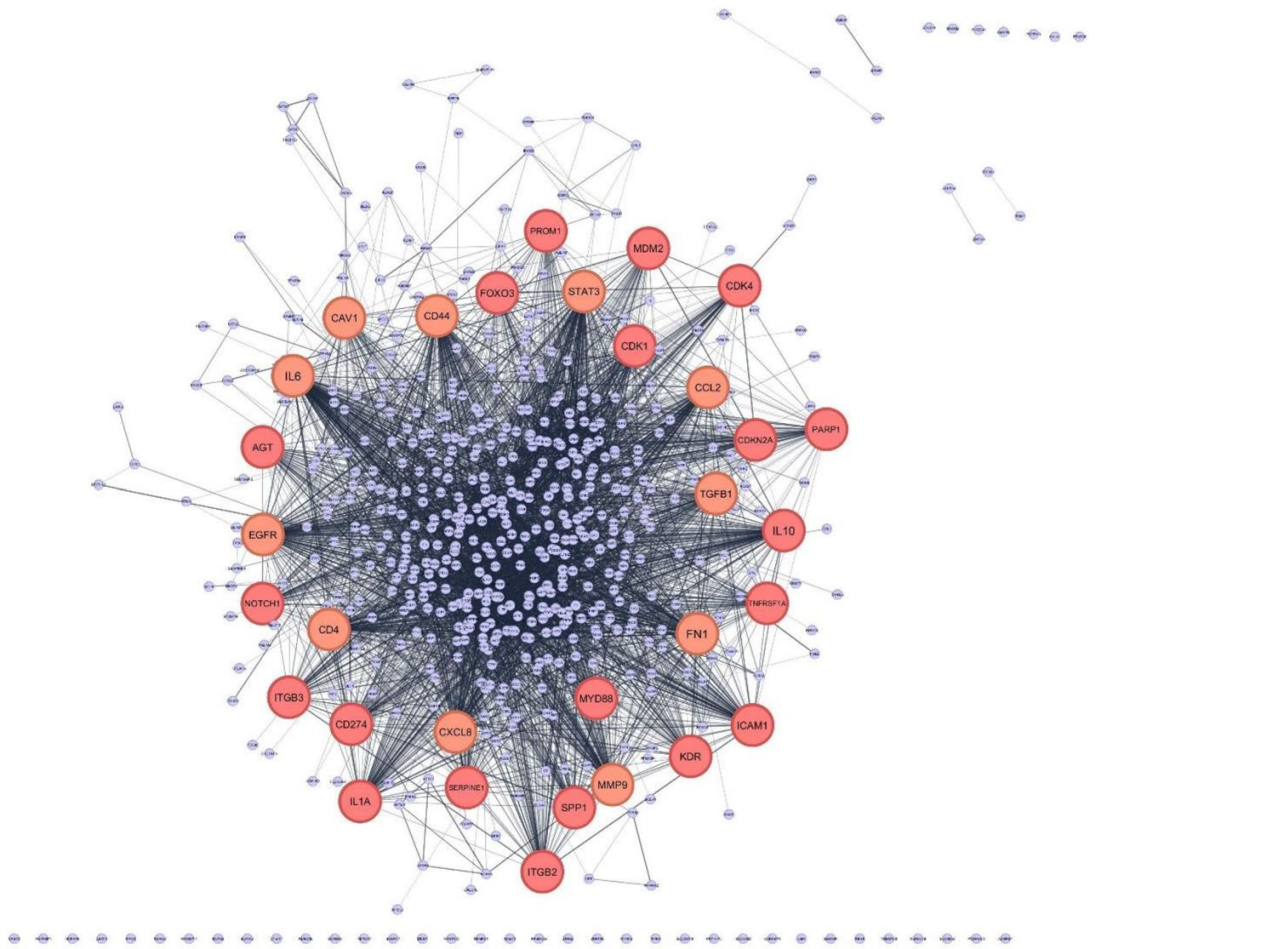
The 613 differentially expressed genes were input into STRING database for PPI network analysis, and achieved a PPI network of 602 nodes and 5570 edges, with PPI enrichment p-value < 1.0 × 10–16. The network was constructed by Cytoscape based on the PPI correlations from the STRING database

**Fig. 3:**
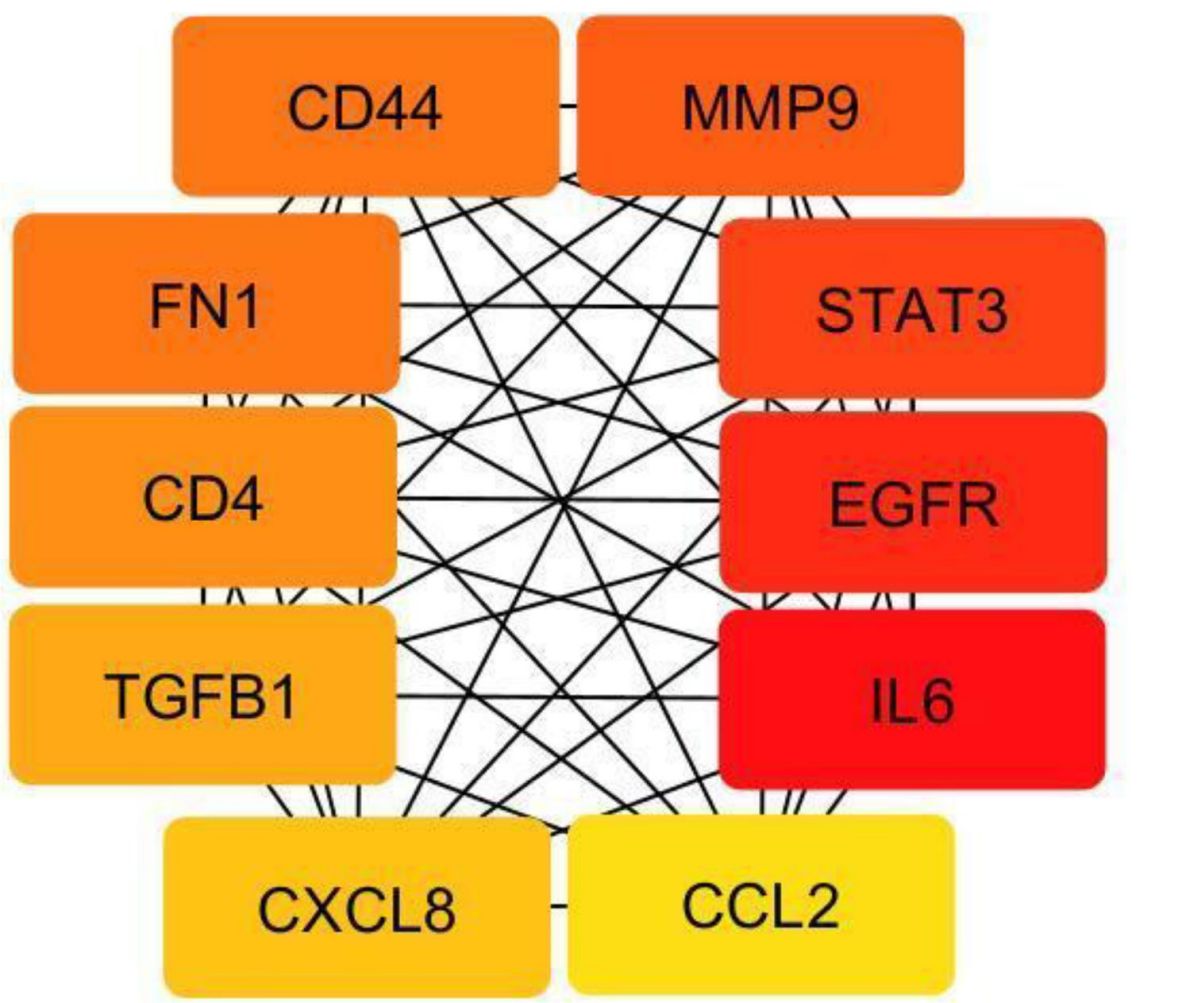
The top 10 genes in the PPI network, in terms of degree ranking, were regarded as hub genes. The node color changes gradually from yellow to red in ascending order according to the degree of the genes

After removing duplicates, 613 genes were included in the bioinformatic analysis (Supplementary File 2). A PPI network was built using the STRING database and Cytoscape application, with an input of 613 genes (Fig. 2). The network was analyzed, and the nodes were ranked based on centrality parameters. The PPI network contains 602 nodes and 5570 edges. Top genes based on the degree value, betweenness centrality, closeness centrality, and stress were selected and organized into 5 groups (Table 2). By considering the degree of connectivity in the PPI network, as described in the materials and methods section, 31 important genes including (IL6, EGFR, STAT3, MMP9, CD44, FN1, CD4, TGFB1, CXCL8, CCL2, IL10, ICAM1, IL1A, CD274, KDR, SPP1, ITGB2, CDKN2A, PARP1, MYD88, AGT, NOTCH1, SERPINE1, TNFRSF1A, CDK1, CAV1, ITGB3, CDK4, FOXO3, MDM2, PROM1), were introduced (Table 2). In addition, the top 10 genes with the highest node degree score were identified as hub genes, as shown in Fig. 3.

**Table 2:** The most 31 important genes related to survival GBM

Gene name	Degree	Stress	Betweenness centrality	Closeness centrality	Radiality	*p*Value of OS in CGGA (*n* = 693)	*p*Value of OS in CGGA (*n* = 325)	*p*Value of OS in GEPIA2
IL6	177	307,218	0.0725	0.5371	0.9951	0.57	0.024	0.074
EGFR	166	438,982	0.1248	0.5387	0.9951	0.28	0.11	0.93
STAT3	142	220,886	0.0470	0.5159	0.9947	0.28	0.039	0.39
MMP9	136	186,362	0.0334	0.5051	0.9945	0.095	0.0016	0.11
CD44	129	190,878	0.0429	0.5046	0.9945	0.095	0.16	0.052
FN1	129	204,432	0.0491	0.5074	0.9945	0.02	0.011	0.028
CD4	127	180,146	0.0444	0.4924	0.9942	0.27	0.045	0.24
TGFB1	120	116,178	0.0206	0.4799	0.9942	0.93	0.099	0.42
CXCL8	118	92,292	0.0166	0.4754	0.9938	NR	NR	0.049
CCL2	113	83,784	0.0131	0.4799	0.9939	0.33	0.0039	0.07
IL10	109	78,812	0.0129	0.4758	0.9938	0.99	0.011	0.26
ICAM1	108	76,042	0.0119	0.4795	0.9939	0.08	0.0016	0.14
IL1A	99	54,768	0.0089	0.4603	0.9934	0.36	0.5	0.53
CD274	83	101,018	0.0236	0.4595	0.9934	0.58	0.061	0.38
KDR	75	66,150	0.0127	0.4599	0.9934	0.71	0.0021	0.98
SPP1	75	67,870	0.0148	0.4645	0.9935	0.25	0.46	0.3
ITGB2	73	64,858	0.0129	0.4439	0.9929	0.25	0.11	0.5
CDKN2A	73	101,996	0.0175	0.4645	0.9935	0.006	0.9	0.55
PARP1	72	123,534	0.0234	0.4641	0.9935	0.81	0.69	0.96
MYD88	71	46,916	0.0093	0.4418	0.9929	0.052	0.00041	0.31
AGT	71	76,346	0.0166	0.4516	0.9931	0.29	0.49	0.71
NOTCH1	69	94,738	0.0208	0.4576	0.9933	0.049	0.061	0.6
SERPINE1	68	47,842	0.0082	0.4486	0.9931	0.094	0.15	0.12
TNFRSF1A	67	44,156	0.0100	0.4439	0.9929	0.24	0.12	0.039
CDK1	65	104,270	0.0188	0.4453	0.9930	0.29	0.62	0.45
CAV1	63	109,384	0.0267	0.4497	0.9931	0.26	0.11	0.44
ITGB3	56	66,744	0.0159	0.4428	0.9929	NR	NR	0.24
CDK4	56	74,876	0.0149	0.4435	0.9929	0.54	0.96	0.43
FOXO3	55	59,820	0.0131	0.4519	0.9934	0.79	0.22	0.71
MDM2	52	68,820	0.0158	0.4482	0.9930	0.36	0.029	0.15
PROM1	48	37,454	0.0085	0.4382	0.9928	0.7	0.97	0.86

OS, overall survival; GEPIA2, Gene Expression Profiling Interactive Analysis 2 ; NR, not result

**Fig. 4:**
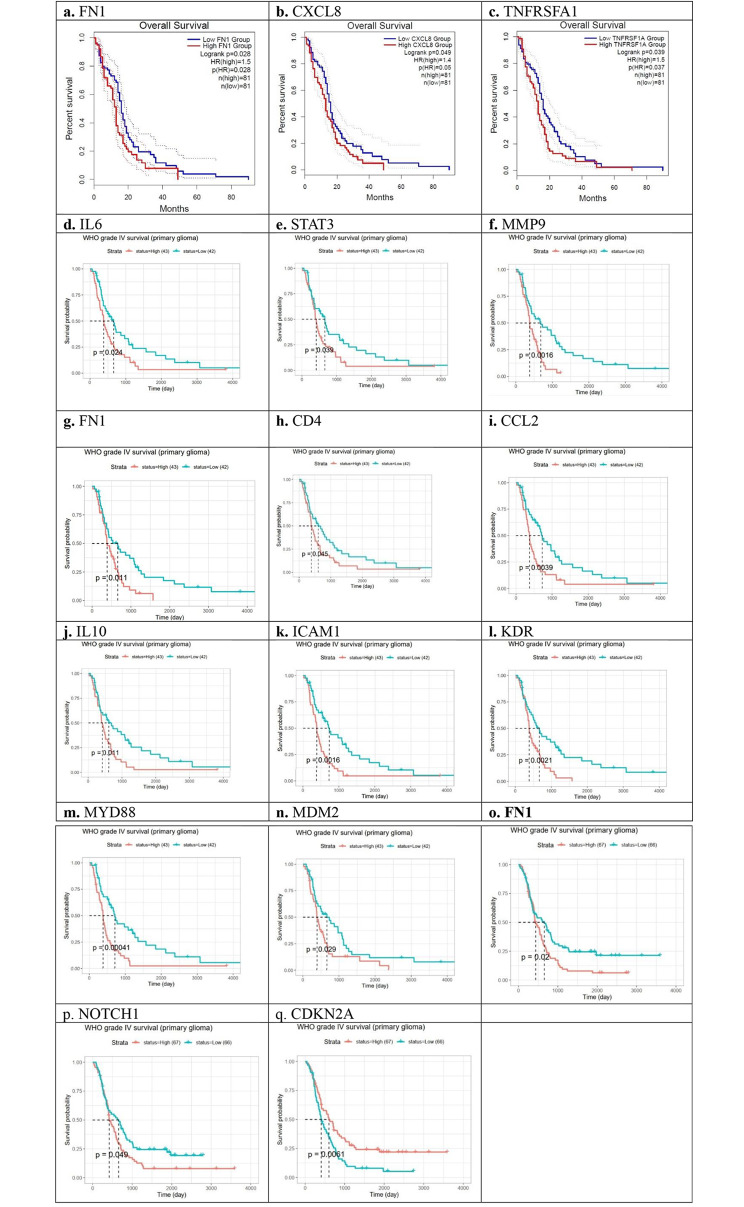
(**a**–**n**) Kaplan-Meier analysis of overall survival for GBM patients in the GEPIA2 using the TCGA cohort (**a**. FN1; **b**. CXCL8; and **c**. TNFRSF1A), the mRNA_seq325 of the CCGA (**d**. IL6; **e**. STAT3; **f**. MMP9; **g**. FN1; **h**. CD4; **i**. CCL2; **j**. IL10; **k**. ICAM1; **l**. KDR; **m**. MYD88; and **n**, MDM2), and the mRNA_seq693 of the CCGA (**o**, FN1, **p**, NOTCH1, **q**, CDKN2A) based on low- and high-expression of genes. The red line represents samples with high expression of the genes, and the blue line represents the samples with low expression of genes. Among 31 genes, *p* < 0.05 was considered to be statistically different

In the validation step as shown in Fig. 4, and Table 2, genes (FN1, CXCL8, and TNFRSF1A) from the GEPIA2 dataset, genes (IL6, STAT3, MMP9, FN1, CD4, CCL2, IL10, ICAM1, KDR, MYD88, MDM2) from the mRNA_seq325 of the CCGA, and genes (FN1, NOTCH1, CDKN2A) from the mRNA_seq693 of the CCGA cohort were associated significantly with overall survival in GBM patients.

The list of the top 31 genes is used as input for computing enrichment. As a result, 1271 GOBP terms were found and 11 GOBP terms were considered. The complete list of significantly enriched GOBP terms and related genes is given in Table 3.

**Table 3:** The top enriched gene ontology biological process terms

GO-term	Description	Count	Adjusted *p*-value	Odds ratio	Genes
GO:0042127	Regulation Of Cell Population Proliferation	14	7.972e-10	21.04	IL10, IL6, IL1A, NOTCH1, EGFR, TGFB1, STAT3, CXCL8, CDK4, CDKN2A, MDM2, KDR, AGT, FN1
GO:0042981	Regulation Of Apoptotic Process	10	0.000002669	13.21	IL10, IL6, EGFR, MMP9, CD44, CDK1, MDM2, AGT, KDR, FOXO3
GO:2,000,045	Regulation Of G1/S Transition Of Mitotic Cell Cycle	3	0.003373	20.67	CDKN2A, CCL2, EGFR
GO:0030334	Regulation Of Cell Migration	8	0.000007457	15.96	EGFR, NOTCH1, TGFB1, STAT3, SERPINE1, FOXO3, KDR, CAV1
GO:0045597	Positive Regulation Of Cell Differentiation	4	0.004627	10.46	IL6, TGFB1, KDR, AGT
GO:0045596	Negative Regulation Of Cell Differentiation	6	0.00001759	23.60	IL6, NOTCH1, TGFB1, ITGB3, CAV1, EGFR
GO:0045765	Regulation Of Angiogenesis	5	0.0002312	19.01	IL1A, IL6, CXCL8, SERPINE1, KDR
GO:0010574	Regulation Of Vascular Endothelial Growth Factor Production	3	0.0001532	92.92	IL1A, IL6, TGFB1
GO:0045687	Positive Regulation Of Glial Cell Differentiation	2	0.002153	91.74	NOTCH1, TGFB1
GO:0048710	Regulation Of Astrocyte Differentiation	2	0.001233	152.95	NOTCH1, IL6
GO:0060251	Regulation of glial cell proliferation	2	0.0007411	229.46	NOTCH1, IL6

## Discussion

To the best of our knowledge, this systematic literature review is the most comprehensive review of gene expression for predicting GBM overall survival outcomes. The most 31 important genes including IL6, EGFR, STAT3, MMP9, CD44, FN1, CD4, TGFB1, CXCL8, CCL2, IL10, ICAM1, IL1A, CD274, KDR, SPP1, ITGB2, CDKN2A, PARP1, MYD88, AGT, NOTCH1, SERPINE1, TNFRSF1A, CDK1, CAV1, ITGB3, CDK4, FOXO3, MDM2, and PROM1, respectively, were considered as candidate biomarkers for GBM survival. Our analyses showed that in fact they all could be considered biomarkers. Nevertheless, based on the search strategy (Supplementary File 1, Table S2.), this review aimed to conduct a comprehensive, systematic literature review to identify all relevant studies that have significantly reported genes related to overall survival in GBM patients. However, some impact reports on this topic might have been missed due to limitations in the search strategy [265–266]. In the study future, given the well-established heterogeneity of GBM, the assessment of the prognostic value of specific genes must be conducted with consideration for GBM molecular subtypes, to ensure a comprehensive understanding of their impact, and would pave the way for precision medicine [266].

Detection of a specific gene expression in GBM tumors may be used to diagnose the existence of a GBM disease or enable clinicians to select the most effective treatment. As there was heterogeneity among the studied genes, bioinformatic analyses were performed to compile these data. The results identified 31 key genes, which had high weight and good topological properties (degree, stress, betweenness, closeness, and radiality) in the pathogenic networks. In addition, these genes were validated by RT-qPCR assays or bioinformatic analysis of datasets. In this study according to 5 typical nodal metrics, we found the most 31 important genes related to the survival of patients with GBM. However, there is currently no consensus on how to use these metrics for the interpretation of biological networks [267]. Therefore, these findings require further investigation.

Identification of survival-associated genes in GBMs has been ongoing over the past decade. However, the gene lists identified by researchers [8–262] differ considerably; only 107 common genes from 720 genes could be identified in studies. These differences can be attributed to two major factors. First, researchers have analyzed GBM datasets from various cohorts worldwide. Second, studies have analyzed different types of datasets, obtained using different approaches such as PCR or next-generation sequencing data. Due to technical limitations and cohorts’ specificity, the expression profiles of similar genes identified from different datasets may be inconsistent.

To improve prognostic and predictive survival power in GBM patients, researchers [69, 91–92, 94–95, 97, 109, 131, 137–138, 144, 147, 172, 186, 189, 195, 197, 200, 222, 242, 251, 253], identified a panel of 2, 3, 4, 6,7, 8, 13, or 14 genes using mRNA expression datasets. They established a risk score model that performed well in survival prediction. High-risk group patients had significantly poorer survival as compared with those in the low-risk group. In this study, AUC for the 1-year overall survival predictions was reported between o.587 [195], to 0.905 [69]. This difference may be due to the use of different databases and cohorts’ specificities. The obtained 31 mRNA panel in this study, is suggested to predict OS in glioblastoma in various cohorts.

The present study showed that ten hub genes (IL6, EGFR, STAT3, MMP9, CD44, FN1, CD4, TGFB1, CXCL8, CCL2) with higher node degree in PPI networks have been predicted to be survival biomarkers for GBM patients and some have been experimentally validated. These hub genes can be offered to the candidate biomarkers of future research for therapeutic targets in patients with GBM. In addition, this study showed that five hub genes (IL6, NOTCH1, TGFB1, EGFR, and KDR) were involved in most of the pathways, and they can be further investigated for biological discoveries. Moreover, we noticed that cell proliferation, apoptotic process, cell migration, and cell differentiation contain many of these genes (see Table 3). In addition, various studies showed that overexpression of these five genes leads to increased cell proliferation and invasion, and inhibition of apoptosis in glioblastoma tumors and was associated with poor patient survival [268–271]. Therefore, these GOBP terms may exert a synergistic effect on the survival of GBM, which could be clues to therapeutic strategies for this disease.

One might inquire about the hub genes obtained from this study. Certainly, various computer modeling algorithms and prediction methods have been and are being developed and used to predict outcomes in medical research. It is noted that each modeling approach has its strengths and weaknesses and there is no best one for all cases [272]. The best modeling approach is uncertain, and may be obtained by combining more than one model, and research in this field continues [272]. By changing the method of prediction, the most important variables will be changed to predict outcomes [272]. We identified two hub gene groups that were associated with overall survival in GBM patients. The ten and five hub genes are ranked by degree and pathways analysis methods, respectively. It is noted that, when a different gene selection criterion is applied, the number of genes in the two top-ranking lists of the two methods will also change [273]. In this study, the algorithms yielded different top-ranking gene lists due to their different approach. Interestingly, the two lists of hub genes have three in common, that were selected as the most important genes for the prediction of survival in methods and can be considered as three hub genes (IL6, TGFB1, and EGFR).

In this study, the five and ten lists of hub genes were selected based on two different methods, therefore, the two groups are different [272–273]. On the other hand, as we all know, the TCGA and the CGGA databases are the world’s largest and most comprehensive gene expression public databases in GBM patients. Hence, these databases were used for validation of our study results. In the validation analysis, we used the GEPIA2, the mRNA_seq325, and the mRNA_seq693 of the CCGA. Only, the FN1 gene was significant in three cohorts. Although the mRNA_seq693 includes more patients with Grade 4 glioma compared to the mRNA_seq325, only three genes were significant compared to eleven genes observed in the other cohort of the CGGA (Table 3). The differences seen between the three databases can be due to the differences in genetics between the different populations.

### Study consistency

Of the 255 manuscripts, all studies were prospective. No randomized trial was found. 107 mRNAs (14.9%; 107/720) were common in all studies. However, a large number of studies have not been validated; hence, there was a lack of high-quality evidence in this study. 124 studies were rated as fair quality; 131 studies were considered to be of high quality. Types of studies and datasets were not consistently reported, resulting in a potential bias.

Among studies in this systematic review, genes with a significant correlation between gene expression and overall survival were considered. Previous studies have found that gene expression levels are associated with prognosis and some genes can be applied to predict the survival risk of GBM patients. However, some studies have conflicts regarding significant differences in gene expression and overall survival. These conflicts seem to depend on the GBM sample size, the heterogeneity of GBM, the datasets used, and the methodologies employed. All the above- mentioned may explain why the validation step did not yield significant results.

### Study quality

The small sample size in PCR-based studies, the high number of single-center studies, various databases, and the high number of studies from China (161/255), the USA (23/155), and India (10/255), which may affect the quality of studies. The heterogeneity of the studies reduced the quality of the data. In some of these datasets and GBM samples, the type and severity of GBM disease were not specified. In addition, some studies lacked validation of their candidate genes in a GBM patient cohort. Therefore, further research with large sample sizes and validation in GBM patients is warranted.

### Strengths, limitations, and future perspectives

To the best of our knowledge, the current study is the first that systematically reviewed published data on gene expression related to the survival of GBM patients. Of note, the major strength of the current systematic review is that bioinformatic analyses were performed, which added new information to the previously studied gene expression on this topic. The findings reported here provide a better view of gene expression biomarkers in predicting the prognosis of patients with GBM.

There are some limitations in our work. Firstly, the search strategy was restricted to the English language literature only, hence, there is a possibility of excluding qualified studies published in other languages. Secondly, the study showed a high level of heterogeneity in the methods used among the included studies. In particular, there were heterogeneities in (1) Variety in disease severity; and (2) Age- and gender-related changes in GBM patients were not considered. Thirdly, the overall survival has been associated with multiple factors such as poor immune response, which was not considered in this study. Additionally, the role of gene expression was not completely clarified in various biological processes and the potential application of these molecules as gene therapies. Hence, future studies are required to clarify the biological roles of the mRNAs to investigate the possibility of their clinical utilization in GBM patients.

## Conclusion

Our review suggests that the current evidence for gene expression associated with GBM survival is highly variable. At present, no clear decisions can be made from this systematic review for application into clinical practice. The key recommendation from this study is that genetic data sharing develops strategies and guidelines in this field that can be used to answer important questions. Moreover, in future a combination of significant genes expression signatures can be applied to identify a powerful and independent predictor for outcome in GBM patients.

### Electronic supplementary material

Below is the link to the electronic supplementary material.

Supplementary Material 1

Supplementary Material 2

Supplementary Material 3

## Data Availability

All data analyzed during this study are included in this published article. GEPIA2 and CGGA databases are publicly available at (http://gepia2.cancer-pku.cn) and (http://www.cgga.org.cn), respectively.

## Abbreviations

GBM: Glioblastoma Multiforme
CNS: Central Nervous System
OS: Overall Survival
TCGA: The Cancer Genome Atlas
CGGA: Chinese Glioma Genome Atlas
GEPIA: Gene Expression Profiling Interactive Analysis
PPI: Protein-Protein Interaction
AUC: Area under curve
GEO: Gene Expression Omnibus
WHO: World Health Organization
