# Supplementary material for: mRNA markers for survival prediction in glioblastoma multiforme patients: a systematic review with bioinformatic analyses
Source: BMC Cancer. 2024 May 21;24:612. doi: 10.1186/s12885-024-12345-z (PMC11106946; doi:10.1186/s12885-024-12345-z)
Supplement: Supplementary file 2 — Supplementary Material 2 [file 12885_2024_12345_MOESM2_ESM.doc]

**mRNA markers for survival prediction in GBM patients: A systematic review with bioinformatic analyses**

Parisa Azimi^1^,MD; Taravat Yazdanian^2^, MD; Abolhassan Ahmadiani ^1^, PhD.

Contents

PRISMA checklist 2

Search strategy 4

NOS quality assessment checklist. 5

**Supplementary Table S1**: PRISMA Checklist

| **Section/topic** | | | **#** | | **Checklist item** | **Reported on page #** |  |
| --- | --- | --- | --- | --- | --- | --- | --- |
| **TITLE** | | | | | |  |  |
| Title | | | 1 | | Identify the report as a systematic review, meta-analysis, or both. | 1 |  |
| **ABSTRACT** | | | | | |  |  |
| Structured summary | | | 2 | | Provide a structured summary including, as applicable: background; objectives; data sources; study eligibility criteria, participants, and interventions; study appraisal and synthesis methods; results; limitations; conclusions and implications of key findings; systematic review registration number. | 2 |  |
| **INTRODUCTION** | | | | | |  |  |
| Rationale | | | 3 | | Describe the rationale for the review in the context of what is already known. | 3-4 |  |
| Objectives | | | 4 | | Provide an explicit statement of questions being addressed with reference to participants, interventions, comparisons, outcomes, and study design (PICOS). | 4 |  |
| **METHODS** | | | | | |  |  |
| Protocol and registration | | 5 | | Indicate if a review protocol exists, if and where it can be accessed (e.g., Web address), and, if available, provide registration information including registration number. | | 4-5 and attached file |  |
| Eligibility criteria | | 6 | | Specify study characteristics (e.g., PICOS, length of follow-up) and report characteristics (e.g., years considered, language, publication status) used as criteria for eligibility, giving rationale. | | 4-5 |  |
| Information sources | | 7 | | Describe all information sources (e.g., databases with dates of coverage, contact with study authors to identify additional studies) in the search and date last searched. | | 4-5 and attached file |  |
| Search | | 8 | | Present full electronic search strategy for at least one database, including any limits used, such that it could be repeated. | | 4-5 and attached file |  |
| Study selection | | 9 | | State the process for selecting studies (i.e., screening, eligibility, included in systematic review, and, if applicable, included in the meta-analysis). | | 4-5 |  |
| Data collection process | | 10 | | Describe method of data extraction from reports (e.g., piloted forms, independently, in duplicate) and any processes for obtaining and confirming data from investigators. | | 4-5 |  |
| Data items | | 11 | | List and define all variables for which data were sought (e.g., PICOS, funding sources) and any assumptions and simplifications made. | | 4-5 |  |
| Risk of bias in individual studies | | 12 | | Describe methods used for assessing risk of bias of individual studies (including specification of whether this was done at the study or outcome level), and how this information is to be used in any data synthesis. | | 5 |  |
| Summary measures | | 13 | | State the principal summary measures (e.g., risk ratio, difference in means). | | 5-6 |  |
| Synthesis of results | | 14 | | Describe the methods of handling data and combining results of studies, if done, including measures of consistency (e.g., I^2^) for each meta-analysis. | | 5-6 |  |
|  | **Section/topic** | **#** | | **Checklist item** | | **Reported on page #** | |
|  | Risk of bias across studies | 15 | | Specify any assessment of risk of bias that may affect the cumulative evidence (e.g., publication bias, selective reporting within studies). | | 5 | |
|  | Additional analyses | 16 | | Describe methods of additional analyses (e.g., sensitivity or subgroup analyses, meta-regression), if done, indicating which were pre-specified. | | 5-6 | |
|  | **RESULTS** | | | | |  | |
|  | Study selection | 17 | | Give numbers of studies screened, assessed for eligibility, and included in the review, with reasons for exclusions at each stage, ideally with a flow diagram. | | Figure 1 | |
|  | Study characteristics | 18 | | For each study, present characteristics for which data were extracted (e.g., study size, PICOS, follow-up period) and provide the citations. | | 6-7 | |
|  | Risk of bias within studies | 19 | | Present data on risk of bias of each study and, if available, any outcome level assessment (see item 12). | | Table 2 | |
|  | Results of individual studies | 20 | | For all outcomes considered (benefits or harms), present, for each study: (a) simple summary data for each intervention group (b) effect estimates and confidence intervals, ideally with a forest plot. | | 7-8 | |
|  | Synthesis of results | 21 | | Present results of each meta-analysis done, including confidence intervals and measures of consistency. | | - | |
|  | Risk of bias across studies | 22 | | Present results of any assessment of risk of bias across studies (see Item 15). | | 7, Table 2 | |
|  | Additional analysis | 23 | | Give results of additional analyses, if done (e.g., sensitivity or subgroup analyses, meta-regression [see Item 16]). | | 7-8 , supplementary file 2. | |
|  | **DISCUSSION** | | | | |  | |
|  | Summary of evidence | 24 | | Summarize the main findings including the strength of evidence for each main outcome; consider their relevance to key groups (e.g., healthcare providers, users, and policy makers). | | 8 | |
|  | Limitations | 25 | | Discuss limitations at study and outcome level (e.g., risk of bias), and at review-level (e.g., incomplete retrieval of identified research, reporting bias). | | 8-11 | |
|  | Conclusions | 26 | | Provide a general interpretation of the results in the context of other evidence, and implications for future research. | | 8-11 | |
|  | **FUNDING** | | | | |  | |
|  | Funding | 27 | | Describe sources of funding for the systematic review and other support (e.g., supply of data); role of funders for the systematic review. | | 12 | |

# Literature Search strategy

Electronic searches were performed using the Scopus, PubMed, Web of Science, and Cochrane Library databases up to August 2023, and written in English. The literature involving all comparative studies were searched, containing the following search terms: “gene expression” or “expressed genes” or “mRNA” or “RNA-Seq” and “survival” or “prognostic” or “biomarker” and “Glioblastoma multiforme (GBM)” or “high-grade glioma”. Search strategy for data bases is given in table 1. Publication date up to 2024/01/02; Field: Title/Abstract.

**Supplementary Table S2**: Search strategy

| **#** | **Data base** |  |
| --- | --- | --- |
|  | **Search strategy** | |
|  | [PubMed](https://www.ncbi.nlm.nih.gov/pubmed?otool=iauflullib&myncbishare=flinlib) | ((Glioblastoma [Title/Abstract]) OR (GBM[Title/Abstract]) OR (high-grade glioma [Title/Abstract])) AND ((mRNA [Title/Abstract]) OR (gene signature [Title/Abstract]) OR (gene expression [Title/Abstract])) AND ((survival [Title/Abstract])) AND ((PCR [Title/Abstract]) OR (Microarray[Title/Abstract]) OR (database [Title/Abstract]) OR (dataset [Title/Abstract]) OR (patient [Title/Abstract]) OR (RNA-seq [Title/Abstract])) Sort by: Publication Date Filters: Publication date up to 2024/01/01; Field: Title/Abstract. 1156 |
|  | [Scopus](http://ezproxy.flinders.edu.au/login?url=https://www.scopus.com/scopus/home.url) | (TITLE-ABS-KEY ( Glioblastoma ) AND TITLE-ABS-KEY ( mRNA ) AND TITLE-ABS-KEY ( survival ) ) Publication date up to 2024/01/02. |
|  | Web of Science | Glioblastoma (All Fields) AND mRNA (All Fields) AND survival (All Fields)  <https://www.webofscience.com/wos/woscc/summary/fb2b77b2-5a43-4a3c-b677-a965e33a9ed4-c1dd6341/relevance/24(overlay:export/exp)> : Publication date up to 2024/01/02. |
|  | Cochrane | (Glioblastoma): all text AND (mRNA): all text AND (survival): all text  (Word variations have been searched) Publication date up to 2024/01/01. |

**Supplementary Table S3.** Check list for quality assessment and scoring of studies based on NOS.

| **Check list** |
| --- |
| *Selection*  1. Is the case definition adequate? (if yes, one star)  2. Representativeness of the sample. Truly representative or somewhat representative? (if yes, one star)  3. How representative was the bioinformatic analysis group in comparison with the validation group, and were assess by mRNA expression? (if yes, one star; no star if the patients were selected only in one group)  4. Use of bioinformatic database analysis and specimen verification as RT-qPCR to identify novel biomarkers predicting survival in GBM tumors. Are both of them used? (if yes, one star) |
| *Comparability*  Comparability of bioinformatic analysis dataset results with other datasets or methods of measurements as RT-qPCR basis of the design or analysis (if yes, two stars; one star was assigned if validation and verification was not reported clearly) |
| *Outcome assessment*  6. Ascertainment of the outcome: clearly defined outcome of mRNA expression, survival analysis, and methods of measurements as RT-qPCR, (yes, two stars for information ascertained; one star if two of this information were not reported)  7. Appropriate statistical analysis: The statistical test used to analyze mRNA and the survival of GBM patients as bioinformatic analysis, RT-qPCR, or microarray were clearly defined and appropriate for bioinformatic analysis group or verification group (if yes, one star; no star was assigned if outcomes were not reported) |

mRNA, messenger RNA; RT-qPCR, Reverse transcription-quantitative polymerase chain reaction.

**Supplementary Table S4:** The quality assessment score for studies under review based on Newcastle-Ottawa Scale (NOS).

|  | **Selection**  (Max-4points) | | | | **Comparability**  (Max-two points) | **Assessment of the outcome**  (Max-three points) | | **Total Score** |
| --- | --- | --- | --- | --- | --- | --- | --- | --- |
| List of studies | Representativeness of the sample | Sample size | Bioinformatic analysis and validation group were assessed in each study | RT-qPCR or microarray were used for validation | The subjects in different outcome groups are comparable, based on the study design or analysis. | Ascertainment of the outcome | Appropriate statistical analysis | case definition adequate |
| Tchirkov et al. [8] | 1 | 1 | 0 | 1 | 1 | 2 | 1 | 7 |
| Santosh et al. [9] | 1 | 1 | 0 | 1 | 1 | 2 | 1 | 7 |
| Kuan et al. [10] | 1 | 1 | 0 | 1 | 1 | 2 | 1 | 7 |
| Ardebili et al. [11] | 1 | 1 | 0 | 1 | 1 | 2 | 1 | 7 |
| Metellus et al. [12] | 1 | 1 | 0 | 1 | 1 | 2 | 1 | 7 |
| Rutkowski et al. [13] | 1 | 1 | 0 | 0 | 1 | 2 | 1 | 6 |
| Jarboe et al. [14] | 1 | 1 | 0 | 0 | 1 | 2 | 1 | 6 |
| Leone et a. [15] | 1 | 1 | 0 | 1 | 1 | 2 | 1 | 7 |
| Xu et al. [16] | 1 | 1 | 0 | 1 | 1 | 2 | 1 | 7 |
| Haapa-Paananen et al. [17] | 1 | 1 | 0 | 0 | 1 | 2 | 1 | 6 |
| Bao et al. [18] | 1 | 1 | 1 | 0 | 2 | 2 | 1 | 8 |
| Arimappamagan et al. [19] | 1 | 1 | 0 | 1 | 1 | 2 | 1 | 7 |
| Fan et al. [20] | 1 | 1 | 0 | 1 | 1 | 2 | 1 | 7 |
| Han et al. [21] | 1 | 1 | 0 | 0 | 1 | 2 | 1 | 6 |
| Kawaguchi et al. [22] | 1 | 1 | 0 | 1 | 1 | 2 | 1 | 7 |
| Bao et al. [23] | 1 | 1 | 1 | 0 | 2 | 2 | 1 | 8 |
| Chen et al. [24] | 1 | 1 | 0 | 0 | 1 | 2 | 1 | 6 |
| Turtoi et al. [25] | 1 | 1 | 0 | 0 | 1 | 2 | 1 | 6 |
| Cheng et al. [26] | 1 | 1 | 0 | 0 | 1 | 2 | 1 | 6 |
| Hua et al. [27] | 1 | 1 | 0 | 1 | 1 | 2 | 1 | 7 |
| Bao et al. [28] | 1 | 1 | 0 | 0 | 1 | 2 | 1 | 6 |
| Xing et al. [29] | 1 | 1 | 0 | 1 | 1 | 2 | 1 | 7 |
| Sibin et al. [30] | 1 | 1 | 0 | 1 | 1 | 2 | 1 | 7 |
| Cai et al. [31] | 1 | 1 | 1 | 0 | 2 | 2 | 1 | 8 |
| Zhou et al. [32] | 1 | 1 | 0 | 0 | 1 | 2 | 1 | 6 |
| Sibin et al. [33] | 1 | 1 | 0 | 1 | 1 | 2 | 1 | 7 |
| Liu et al. [34] | 1 | 1 | 0 | 0 | 1 | 2 | 1 | 6 |
| Frei et al. [35] | 1 | 1 | 0 | 1 | 1 | 2 | 1 | 7 |
| Bache et al. [36] | 1 | 1 | 0 | 1 | 1 | 2 | 1 | 7 |
| Delic et al. [37] | 1 | 1 | 0 | 1 | 1 | 2 | 1 | 7 |
| Stegen et al. [38] | 1 | 1 | 0 | 0 | 1 | 2 | 1 | 6 |
| Zhang et al. [39] | 1 | 1 | 0 | 0 | 1 | 2 | 1 | 6 |
| Cheng et al. [40] | 1 | 1 | 1 | 0 | 2 | 2 | 1 | 8 |
| Li et al. [41] | 1 | 1 | 1 | 0 | 2 | 2 | 1 | 8 |
| Gao et al. [42] | 1 | 1 | 1 | 1 | 2 | 2 | 1 | 9 |
| Lin et al. [43] | 1 | 1 | 0 | 0 | 1 | 2 | 1 | 6 |
| Wang et al. [44] | 1 | 1 | 1 | 0 | 2 | 2 | 1 | 8 |
| Nduom et al. [45] | 1 | 1 | 0 | 0 | 1 | 2 | 1 | 6 |
| Bayin et al. [46] | 1 | 1 | 0 | 0 | 1 | 2 | 1 | 6 |
| Cheng et a. [47] | 1 | 1 | 1 | 0 | 2 | 2 | 1 | 8 |
| Wang et al. [48] | 1 | 1 | 1 | 0 | 2 | 2 | 1 | 8 |
| Steponaitis et al. [49] | 1 | 1 | 0 | 1 | 1 | 2 | 1 | 7 |
| Sun et al. [50] | 1 | 1 | 1 | 0 | 2 | 2 | 1 | 8 |
| Kolodziej et al. [51] | 1 | 1 | 0 | 1 | 1 | 2 | 1 | 7 |
| Codó et al. [52] | 1 | 1 | 1 | 0 | 2 | 2 | 1 | 8 |
| Wang et al. [53] | 1 | 1 | 1 | 0 | 2 | 2 | 1 | 8 |
| Murnyák et al. [54] | 1 | 1 | 1 | 0 | 2 | 2 | 1 | 8 |
| Xu et al. [55] | 1 | 1 | 0 | 0 | 1 | 2 | 1 | 6 |
| Zhu et al. [56] | 1 | 1 | 0 | 0 | 1 | 2 | 1 | 6 |
| Ohtaki et al. [57] | 1 | 1 | 0 | 1 | 1 | 2 | 1 | 7 |
| Zhai et al. [58] | 1 | 1 | 0 | 0 | 1 | 2 | 1 | 6 |
| Soni et al. [59] | 1 | 1 | 0 | 1 | 1 | 2 | 1 | 7 |
| Haynes et al. [60] | 1 | 1 | 0 | 0 | 1 | 2 | 1 | 6 |
| Huang et al. [61] | 1 | 1 | 1 | 0 | 2 | 2 | 1 | 8 |
| Kim et al. [62] | 1 | 1 | 0 | 0 | 1 | 2 | 1 | 6 |
| Wang et al. [63] | 1 | 1 | 0 | 0 | 1 | 2 | 1 | 6 |
| Han et al. [64] | 1 | 1 | 1 | 0 | 2 | 2 | 1 | 8 |
| Feldheim et al. [65] | 1 | 1 | 0 | 1 | 1 | 2 | 1 | 7 |
| Jia et al. [66] | 1 | 1 | 1 | 0 | 2 | 2 | 1 | 8 |
| Yue et al. [67] | 1 | 1 | 0 | 0 | 1 | 2 | 1 | 6 |
| Wang et al. [68] | 1 | 1 | 1 | 0 | 2 | 2 | 1 | 8 |
| Xu et al. [69] | 1 | 1 | 1 | 0 | 2 | 2 | 1 | 8 |
| Roy et al. [70] | 1 | 1 | 0 | 0 | 1 | 2 | 1 | 6 |
| Chen et al. [71] | 1 | 1 | 1 | 0 | 2 | 2 | 1 | 8 |
| Shu et al. [72] | 1 | 1 | 1 | 0 | 2 | 2 | 1 | 8 |
| Liu et al. [73] | 1 | 1 | 1 | 0 | 2 | 2 | 1 | 8 |
| Zhang et al. [74] | 1 | 1 | 1 | 0 | 2 | 2 | 1 | 8 |
| Gilder et al. [75] | 1 | 1 | 0 | 0 | 1 | 2 | 1 | 6 |
| Takashima et al. [76] | 1 | 1 | 0 | 0 | 1 | 2 | 1 | 6 |
| Han et al. [77] | 1 | 1 | 0 | 0 | 1 | 2 | 1 | 6 |
| Vasaikar et al. [78] | 1 | 1 | 0 | 0 | 1 | 2 | 1 | 6 |
| Cheng et al. [79] | 1 | 1 | 0 | 0 | 1 | 2 | 1 | 6 |
| Zhang et al. [80] | 1 | 1 | 0 | 0 | 1 | 2 | 1 | 6 |
| Breznik et al. [81] | 1 | 1 | 0 | 0 | 1 | 2 | 1 | 6 |
| Cho et al. [82] | 1 | 1 | 0 | 1 | 1 | 2 | 1 | 7 |
| Gao et al. [83] | 1 | 1 | 0 | 0 | 1 | 2 | 1 | 6 |
| Zhou et al. [84] | 1 | 1 | 1 | 0 | 2 | 2 | 1 | 8 |
| Guan et al. [85] | 1 | 1 | 0 | 0 | 1 | 2 | 1 | 6 |
| Zhang et al. [86] | 1 | 1 | 1 | 0 | 2 | 2 | 1 | 8 |
| Zhong et al. [87] | 1 | 1 | 0 | 0 | 1 | 2 | 1 | 6 |
| Du et al. [88] | 1 | 1 | 1 | 0 | 2 | 2 | 1 | 8 |
| Wang et al. [89] | 1 | 1 | 1 | 1 | 2 | 2 | 1 | 9 |
| Werner et al. [90] | 1 | 1 | 1 | 0 | 2 | 2 | 1 | 8 |
| Guo et al. [91] | 1 | 1 | 1 | 0 | 2 | 2 | 1 | 8 |
| Yin et al. [92] | 1 | 1 | 1 | 0 | 2 | 2 | 1 | 8 |
| Cao et al. [93] | 1 | 1 | 1 | 0 | 2 | 2 | 1 | 8 |
| Zuo et al. [94] | 1 | 1 | 1 | 0 | 2 | 2 | 1 | 8 |
| Cheng et al. [95] | 1 | 1 | 1 | 0 | 2 | 2 | 1 | 8 |
| Hsu et al. [96] | 1 | 1 | 1 | 0 | 2 | 2 | 1 | 8 |
| Wang et al. [97] | 1 | 1 | 1 | 0 | 2 | 2 | 1 | 8 |
| Yuan et al. [98] | 1 | 1 | 0 | 1 | 1 | 2 | 1 | 7 |
| Masiulionytė et al. [99] | 1 | 1 | 0 | 1 | 1 | 2 | 1 | 7 |
| Morelli et al. [100] | 1 | 1 | 0 | 1 | 1 | 2 | 1 | 7 |
| Kruthika et al. [101] | 1 | 1 | 1 | 0 | 2 | 2 | 1 | 8 |
| Liu et al. [102] | 1 | 1 | 1 | 0 | 2 | 2 | 1 | 8 |
| Yang et al.[103] | 1 | 1 | 1 | 0 | 2 | 2 | 1 | 8 |
| Liu et al.[104] | 1 | 1 | 1 | 0 | 2 | 2 | 1 | 8 |
| Zhou et al.[105] | 1 | 1 | 0 | 0 | 1 | 2 | 1 | 6 |
| Wang et al.[106] | 1 | 1 | 0 | 0 | 1 | 2 | 1 | 6 |
| Fu et al. [107] | 1 | 1 | 0 | 0 | 1 | 2 | 1 | 6 |
| Hasan et al. [108] | 1 | 1 | 0 | 0 | 1 | 2 | 1 | 6 |
| Ye et al. [109] | 1 | 1 | 1 | 1 | 2 | 2 | 1 | 8 |
| Zeng et al. [110] | 1 | 1 | 0 | 0 | 1 | 2 | 1 | 6 |
| Zhang et al. [111] | 1 | 1 | 1 | 1 | 2 | 2 | 1 | 9 |
| Chen et al. [112] | 1 | 1 | 0 | 0 | 1 | 2 | 1 | 6 |
| Tang et al. [113] | 1 | 1 | 0 | 0 | 1 | 2 | 1 | 6 |
| Waterset al. [114] | 1 | 1 | 0 | 0 | 1 | 2 | 1 | 6 |
| Yuan et al. [115] | 1 | 1 | 0 | 0 | 1 | 2 | 1 | 6 |
| Yang et al. [116] | 1 | 1 | 0 | 0 | 1 | 2 | 1 | 6 |
| Zeng et al. [117] | 1 | 1 | 0 | 0 | 1 | 2 | 1 | 6 |
| Xu et al. [118] | 1 | 1 | 0 | 0 | 1 | 2 | 1 | 6 |
| Fernández-García et al. [119] | 1 | 1 | 0 | 0 | 1 | 2 | 1 | 6 |
| Luo et al. [120] | 1 | 1 | 1 | 0 | 2 | 2 | 1 | 8 |
| Ji et al. [121] | 1 | 1 | 0 | 0 | 1 | 2 | 1 | 6 |
| Dong et al. [122] | 1 | 1 | 0 | 0 | 1 | 2 | 1 | 6 |
| Wang et al. [123] | 1 | 1 | 0 | 1 | 1 | 2 | 1 | 7 |
| Wang et al. [124] | 1 | 1 | 0 | 0 | 1 | 2 | 1 | 6 |
| Steponaitis et al. [125] | 1 | 1 | 0 | 1 | 1 | 2 | 1 | 7 |
| Wang et al. [126] | 1 | 1 | 0 | 0 | 1 | 2 | 1 | 6 |
| Zhang et al. [127] | 1 | 1 | 0 | 0 | 1 | 2 | 1 | 6 |
| Jovčevska et al. [128] | 1 | 1 | 0 | 0 | 1 | 2 | 1 | 6 |
| Komaki et al. [129] | 1 | 1 | 0 | 1 | 1 | 2 | 1 | 7 |
| Sharma et al. [130] | 1 | 1 | 0 | 0 | 1 | 2 | 1 | 6 |
| Prasad et al. [131] | 1 | 1 | 1 | 0 | 2 | 2 | 1 | 8 |
| Vachher et al. [132] | 1 | 1 | 1 | 0 | 2 | 2 | 1 | 8 |
| Nesterova et al. [133] | 1 | 1 | 1 | 0 | 2 | 2 | 1 | 8 |
| Haddad et al. [134] | 1 | 1 | 1 | 0 | 2 | 2 | 1 | 8 |
| Sun et al. [135] | 1 | 1 | 1 | 0 | 2 | 2 | 1 | 8 |
| Du et al. [136] | 1 | 1 | 1 | 1 | 2 | 2 | 1 | 9 |
| Pan et al. [137] | 1 | 1 | 1 | 0 | 2 | 2 | 1 | 8 |
| Valiulyte et al. [138] | 1 | 1 | 1 | 1 | 2 | 2 | 1 | 9 |
| Liu et al. [139] | 1 | 1 | 1 | 0 | 2 | 2 | 1 | 8 |
| Zhu et al. [140] | 1 | 1 | 0 | 0 | 1 | 2 | 1 | 6 |
| Faried et al. [141] | 1 | 1 | 1 | 0 | 2 | 2 | 1 | 8 |
| Hu et al. [142] | 1 | 1 | 0 | 0 | 1 | 2 | 1 | 6 |
| Li et al. [143] | 1 | 1 | 1 | 0 | 2 | 2 | 1 | 8 |
| Yang et al. [144] | 1 | 1 | 0 | 0 | 1 | 2 | 1 | 6 |
| Yarmishyn et al. [145] | 1 | 1 | 0 | 0 | 1 | 2 | 1 | 6 |
| Gan et al. [146] | 1 | 1 | 0 | 1 | 1 | 2 | 1 | 7 |
| Peng et al. [147] | 1 | 1 | 0 | 0 | 1 | 2 | 1 | 6 |
| Song et al. [148] | 1 | 1 | 0 | 0 | 1 | 2 | 1 | 6 |
| Yang et al. [149] | 1 | 1 | 0 | 0 | 1 | 2 | 1 | 6 |
| Wu et al. [150] | 1 | 1 | 0 | 0 | 1 | 2 | 1 | 6 |
| Shi et al. [151] | 1 | 1 | 0 | 0 | 1 | 2 | 1 | 6 |
| Mao et al. [152] | 1 | 1 | 0 | 0 | 1 | 2 | 1 | 6 |
| Fuentes-Fayos ET AL. [153] | 1 | 1 | 1 | 1 | 2 | 2 | 1 | 9 |
| Miao et al. [154] | 1 | 1 | 0 | 0 | 1 | 2 | 1 | 6 |
| Chi et al. [155] | 1 | 1 | 0 | 0 | 1 | 2 | 1 | 6 |
| Kao et al. [156] | 1 | 1 | 1 | 1 | 2 | 2 | 1 | 9 |
| Yi et al. [157] | 1 | 1 | 1 | 0 | 2 | 2 | 1 | 8 |
| Geraldo et al. [158] | 1 | 1 | 1 | 0 | 2 | 2 | 1 | 8 |
| Fang et al. [159] | 1 | 1 | 1 | 0 | 2 | 2 | 1 | 8 |
| Jiang et al. [160] | 1 | 1 | 1 | 0 | 2 | 2 | 1 | 8 |
| Wang et al. [161] | 1 | 1 | 1 | 0 | 2 | 2 | 1 | 8 |
| Maimaiti et al. [162] | 1 | 1 | 0 | 0 | 1 | 2 | 1 | 6 |
| Wang et al. [163] | 1 | 1 | 0 | 0 | 1 | 2 | 1 | 6 |
| Zhao et al. [164] | 1 | 1 | 1 | 0 | 2 | 2 | 1 | 8 |
| Morrison et al. [165] | 1 | 1 | 1 | 0 | 2 | 2 | 1 | 8 |
| Xu et al. [166] | 1 | 1 | 1 | 0 | 2 | 2 | 1 | 8 |
| Jeong et al. [167] | 1 | 1 | 1 | 0 | 2 | 2 | 1 | 8 |
| Cao et al. [168] | 1 | 1 | 1 | 1 | 2 | 2 | 1 | 9 |
| Cheng et al. [169] | 1 | 1 | 1 | 0 | 2 | 2 | 1 | 8 |
| Hu et al. [170] | 1 | 1 | 0 | 0 | 1 | 2 | 1 | 6 |
| Phan et al. [171] | 1 | 1 | 0 | 0 | 1 | 2 | 1 | 6 |
| Hu et al. [172] | 1 | 1 | 1 | 0 | 2 | 2 | 1 | 8 |
| Lan et al. [173] | 1 | 1 | 1 | 0 | 2 | 2 | 1 | 8 |
| Łysiak et al. [174] | 1 | 1 | 0 | 0 | 1 | 2 | 1 | 6 |
| Chen et al. [175] | 1 | 1 | 0 | 0 | 1 | 2 | 1 | 6 |
| Hsieh et al. [176] | 1 | 1 | 0 | 0 | 1 | 2 | 1 | 6 |
| Shi et al. [177] | 1 | 1 | 0 | 0 | 1 | 2 | 1 | 6 |
| Yang et al. [178] | 1 | 1 | 0 | 0 | 1 | 2 | 1 | 6 |
| Zhang et al. [179] | 1 | 1 | 0 | 0 | 1 | 2 | 1 | 6 |
| Urbantat et al. [180] | 1 | 1 | 0 | 0 | 1 | 2 | 1 | 6 |
| Krassnig et al. [181] | 1 | 1 | 0 | 0 | 1 | 2 | 1 | 6 |
| Flor et al. [182] | 1 | 1 | 0 | 0 | 1 | 2 | 1 | 6 |
| Cocola et al. [183] | 1 | 1 | 0 | 0 | 1 | 2 | 1 | 6 |
| Cheng et al. [184] | 1 | 1 | 0 | 0 | 1 | 2 | 1 | 6 |
| Liu et al. [185] | 1 | 1 | 1 | 0 | 2 | 2 | 1 | 8 |
| Li et al. [186] | 1 | 1 | 1 | 0 | 2 | 2 | 1 | 8 |
| Ye et al. [187] | 1 | 1 | 1 | 0 | 2 | 2 | 1 | 8 |
| Zhou et al. [188] | 1 | 1 | 0 | 0 | 1 | 2 | 1 | 6 |
| Tu et al. [189] | 1 | 1 | 1 | 0 | 2 | 2 | 1 | 8 |
| Zhou et al. [190] | 1 | 1 | 0 | 0 | 1 | 2 | 1 | 6 |
| Moriconi et al. [191] | 1 | 1 | 0 | 0 | 1 | 2 | 1 | 6 |
| Stanke et al. [192] | 1 | 1 | 0 | 0 | 1 | 2 | 1 | 6 |
| Redekar et al. [193] | 1 | 1 | 1 | 0 | 2 | 2 | 1 | 8 |
| Liao et al. [194] | 1 | 1 | 1 | 0 | 2 | 2 | 1 | 8 |
| Zhao et al. [195] | 1 | 1 | 1 | 0 | 2 | 2 | 1 | 8 |
| Nimbalkar et al. [196] | 1 | 1 | 1 | 1 | 2 | 2 | 1 | 9 |
| Wu et al. [197] | 1 | 1 | 1 | 0 | 2 | 2 | 1 | 8 |
| Zhang et al. [198] | 1 | 1 | 0 | 0 | 1 | 2 | 1 | 6 |
| Pan et al. [199] | 1 | 1 | 1 | 0 | 2 | 2 | 1 | 8 |
| Y uan et al. [200] | 1 | 1 | 1 | 0 | 2 | 2 | 1 | 8 |
| Jin et al. [201] | 1 | 1 | 1 | 0 | 2 | 2 | 1 | 8 |
| Li et al. [202] | 1 | 1 | 1 | 0 | 2 | 2 | 1 | 8 |
| Santoni et al. [203] | 1 | 1 | 0 | 1 | 1 | 2 | 1 | 7 |
| Dubois et al. [204] | 1 | 1 | 1 | 0 | 2 | 2 | 1 | 8 |
| Trivedi et al. [205] | 1 | 1 | 0 | 1 | 1 | 2 | 1 | 7 |
| Xiong et al. [206] | 1 | 1 | 1 | 0 | 2 | 2 | 1 | 8 |
| Zhang et al. [207] | 1 | 1 | 0 | 0 | 1 | 2 | 1 | 6 |
| Wang et al. [208] | 1 | 1 | 0 | 0 | 1 | 2 | 1 | 6 |
| Park et al. [209] | 1 | 1 | 0 | 0 | 1 | 2 | 1 | 6 |
| Moreno et al. [210] | 1 | 1 | 0 | 0 | 1 | 2 | 1 | 6 |
| Akçay et al. [211] | 1 | 1 | 0 | 0 | 1 | 2 | 1 | 6 |
| Liu et al. [212] | 1 | 1 | 0 | 0 | 1 | 2 | 1 | 6 |
| Lin et al. [213] | 1 | 1 | 0 | 0 | 1 | 2 | 1 | 6 |
| Güven et al. [214] | 1 | 1 | 1 | 0 | 2 | 2 | 1 | 8 |
| Zheng et al. [215] | 1 | 1 | 0 | 0 | 1 | 2 | 1 | 6 |
| Liu et al. [216] | 1 | 1 | 0 | 1 | 1 | 2 | 1 | 7 |
| Lin et al. [217] | 1 | 1 | 0 | 0 | 1 | 2 | 1 | 6 |
| Cui et al. [218] | 1 | 1 | 1 | 0 | 2 | 2 | 1 | 8 |
| Gao et al. [219] | 1 | 1 | 1 | 0 | 2 | 2 | 1 | 8 |
| Han et al. [220] | 1 | 1 | 0 | 0 | 1 | 2 | 1 | 6 |
| Wei et al. [221] | 1 | 1 | 0 | 0 | 1 | 2 | 1 | 6 |
| Vedunova et al. [222] | 1 | 1 | 1 | 0 | 2 | 2 | 1 | 8 |
| Zhang et al. [223] | 1 | 1 | 0 | 0 | 1 | 2 | 1 | 6 |
| Zhu et al. [224] | 1 | 1 | 0 | 0 | 1 | 2 | 1 | 6 |
| Chen et al. [225] | 1 | 1 | 0 | 0 | 1 | 2 | 1 | 6 |
| Xie et al. [226] | 1 | 1 | 0 | 0 | 1 | 2 | 1 | 6 |
| Fuentes-Fayos et al. [227] | 1 | 1 | 0 | 0 | 1 | 2 | 1 | 6 |
| Guda et al. [228] | 1 | 1 | 0 | 0 | 1 | 2 | 1 | 6 |
| Du et al. [229] | 1 | 1 | 0 | 0 | 1 | 2 | 1 | 6 |
| Phillips et al. [230] | 1 | 1 | 0 | 0 | 1 | 2 | 1 | 6 |
| Li et al. [231] | 1 | 1 | 0 | 0 | 1 | 2 | 1 | 6 |
| Li et al. [232] | 1 | 1 | 0 | 0 | 1 | 2 | 1 | 6 |
| Guan et al. [233] | 1 | 1 | 1 | 0 | 2 | 2 | 1 | 8 |
| Guo et al. [234] | 1 | 1 | 1 | 0 | 2 | 2 | 1 | 8 |
| Wang et al. [235] | 1 | 1 | 0 | 0 | 1 | 2 | 1 | 6 |
| Wan et al. [236 | 1 | 1 | 1 | 0 | 2 | 2 | 1 | 8 |
| Gao et al. [237] | 1 | 1 | 0 | 0 | 1 | 2 | 1 | 6 |
| Liu et al. [238] | 1 | 1 | 1 | 0 | 2 | 2 | 1 | 8 |
| Chen et al. [239] | 1 | 1 | 0 | 1 | 1 | 2 | 1 | 7 |
| Zheng et al. [240] | 1 | 1 | 0 | 0 | 1 | 2 | 1 | 6 |
| Yoon et al. [241] | 1 | 1 | 0 | 0 | 1 | 2 | 1 | 6 |
| Danget al. [242] | 1 | 1 | 1 | 0 | 2 | 2 | 1 | 8 |
| Singh et al. [243] | 1 | 1 | 0 | 0 | 1 | 2 | 1 | 6 |
| Li et al. [244] | 1 | 1 | 1 | 0 | 2 | 2 | 1 | 8 |
| Ali et al. [245] | 1 | 1 | 0 | 0 | 1 | 2 | 1 | 6 |
| Jia et al. [246] | 1 | 1 | 0 | 0 | 1 | 2 | 1 | 6 |
| Ren et al. [247] | 1 | 1 | 0 | 0 | 1 | 2 | 1 | 6 |
| Zheng et al. [248] | 1 | 1 | 0 | 0 | 1 | 2 | 1 | 6 |
| Ma et al. [249] | 1 | 1 | 1 | 0 | 2 | 2 | 1 | 8 |
| Gao et al. [250] | 1 | 1 | 0 | 0 | 1 | 2 | 1 | 6 |
| Chen et al. [251] | 1 | 1 | 1 | 0 | 2 | 2 | 1 | 8 |
| Ye et al. [252] | 1 | 1 | 0 | 0 | 1 | 2 | 1 | 6 |
| Zhu et al. [253] | 1 | 1 | 1 | 0 | 2 | 2 | 1 | 8 |
| Li et al. [254] | 1 | 1 | 0 | 0 | 1 | 2 | 1 | 6 |
| Lin et al. [255] | 1 | 1 | 0 | 0 | 1 | 2 | 1 | 6 |
| Hu et al. [256] | 1 | 1 | 0 | 0 | 1 | 2 | 1 | 6 |
| Ding et al. [257] | 1 | 1 | 0 | 0 | 1 | 2 | 1 | 6 |
| Le et al. [258] | 1 | 1 | 0 | 0 | 1 | 2 | 1 | 6 |
| Wang et al. [259] | 1 | 1 | 0 | 0 | 1 | 2 | 1 | 6 |
| Wu et al. [260] | 1 | 1 | 1 | 0 | 2 | 2 | 1 | 8 |
| Zhu et al. [261] | 1 | 1 | 0 | 0 | 1 | 2 | 1 | 6 |
| Azimi et al. [262] | 1 | 1 | 1 | 1 | 2 | 2 | 1 | 9 |

Wells GA, Shea B, O'Connell D et al. The Newcastle-Ottawa Scale (NOS) for assessing the quality of nonrandomized studies in meta-analyses. <http://www.ohri.ca/> programs/clinical_epidemiology/nosgen.pdf. Accessed on May 11, 2020.
