# Supplementary material for: mRNA markers for survival prediction in glioblastoma multiforme patients: a systematic review with bioinformatic analyses
Source: BMC Cancer. 2024 May 21;24:612. doi: 10.1186/s12885-024-12345-z (PMC11106946; doi:10.1186/s12885-024-12345-z)
Supplement: Supplementary file 3 — Supplementary Material 3 [file 12885_2024_12345_MOESM3_ESM.docx]

**Table 2:** Characteristics of included studies and quality evaluation

|  |  |  | Participants | | | Gene database | | Expression of mRNA in GBM | | |  | |  | |  |
| --- | --- | --- | --- | --- | --- | --- | --- | --- | --- | --- | --- | --- | --- | --- | --- |
|  |  |  | Tissue analysis | GBM | |  |  |  |  | |  | |  | |  |
| Author(s) [Ref.] | Year | Country | Method | M:F ratio | n, Mean age | Training/or selected from Gene Expression | Validation | High expression (OS, Short/Long) | | Low expression (OS, Short/Long) | | OS | | AUC for gene panel | NOS |
| Tchirkov et al. [8] | 2003 | France | RT-qPCR | NR | 43, NR | - | - | TERT (S) | | - | | S | | - | 7 |
| Santosh et al. [9] | 2010 | India | RT-qPCR | NR | 137, NR | - | - | IGFBP-3 (S) | | - | | S | | - | 7 |
| Kuan et al. [10] | 2010 | USA | RT-qPCR | NR | 67, NR | - | - | MRP3 (S) | | - | | S | | - | 7 |
| Ardebili et al. [11] | 2011 | Slovenia | RT-qPCR | 2 | 24, 60 | - | - | CD133(S) | | - | | S | | - | 7 |
| Metellus et al. [12] | 2011 | France | RT-qPCR | 1.3 | 32, 49 | - | - | PROM1(S) ,PPP6C (S) | | CACNA2D3, PPP2R2B, SIK, MAST3 (all shorted survival) | | S | | - | 7 |
| Rutkowski et al. [13] | 2011 | USA | - | - | - | TCGA | - | EMR2 (S) | | - | | S | |  | 6 |
| Jarboe et al. [14] | 2012 | USA | - | - | - | TCGA, REMBRANDT | - | MARCKS (L) | | - | | L | |  | 6 |
| Leone et a. [15] | 2012 | Spain | RT-qPCR | 1.3 | 48, 60.1 | - | - | CD133(S) | | - | | S | | - | 7 |
| Xu et al. [16] | 2012 | China | RT-qPCR | NR | 30, NR | - | - | BSP (S) | | - | | S | | - | 7 |
| Haapa-Paananen et al. [17] | 2012 | Finland | - | - | - | TCGA | - | HES6 (L) | | - | | L | | - | 6 |
| Bao et al. [18] | 2013 | China | - | - | - | TCGA | REMBRANDT, GSE16011 | KCNF1, ALCAM, LHX6, CST4, SPATA6, MEOX2, AMIGO2, TNFAIP6, PCDHGC3, BCAP29, C13orf18, LAMB4, SIGLEC9, ITPKC, EFNA5, ATP1B1, AMOT, RGS6, MYO1D, BRF2, FLJ21963, CHD3, PLEKHA4, HUS1, PTPRK, AGTPBP1, ARNT, ZNF492, SLC38A1, CDC42EP2, C7orf26, KCNE4, RORA, VRK3, LZTS1, PDE4B, C1orf176, FXYD5, ZNF576, ZNF593, PDLIM1, RPS6KA5. (NR), STK17A, NPAS3, MGST3, FOXO4, GTF2A1, AP4M1, NANS, FKRP, FZD7, ALKBH4, TPBG, FBXO7, MNX1, LFNG, SLC4A4, SEMA6D, TMC6 (NR) | | | | NR | | - | 8 |
| Arimappamagan et al. [19] | 2013 | India | RT-qPCR | NR | 123, NR | TCGA | - | SOD2, EGFR, AGT, CHI3L1, CCL2, MBP, CPE, OLFM1, PACSIN1, SNCA (all shorted survival); | | IGFBPL1, MCF2, CALCRL, TOP2A (all longer survival) | | S,L | | - | 7 |
| Fan et al. [20] | 2013 | China | RT-qPCR | 1.9 | 119, NR | - | - | SLC7A7 (S) | | - | | S | | - | 7 |
| Han et al. [21] | 2013 | China | - | - | - | CGGA | - | SATB1 (S) | | - | | S | | - | 6 |
| Kawaguchi et al. [22] | 2013 | Japan | RT-qPCR | 1.7 | 32, NR | TCGA, and three external data sets [22] | - | ASF1A, ITGA7, AFTPH (Gene panel; L) | | - | | L | | - | 7 |
| Bao et al. [23] | 2013 | China | - | - | - | TCGA | CGGA, Rembrandt | BMP4 (S) | | - | | S | | - | 8 |
| Chen et al. [24] | 2014 | China | - | - | - | TCGA | - | TAPBPL, SERPINB9, RCAN1, TMBIM4, JUNB, IL4R, LY75 (all shorted survival) | | MGST3 (S) | | S | | - | 6 |
| Turtoi et al. [25] | 2014 | China | - | - | - | REMBRANDT | - | COL6A1 (S) | | - | | S | | - | 6 |
| Cheng et al. [26] | 2014 | China | - | - | - | GDS1815/219787_s_at/ECT2 | - | ECT2 (S) | | - | | S | | - | 6 |
| Hua et al. [27] | 2014 | China | RT-qPCR | NR | 24, NR | - | - | MCM2, MCM3, MCM7 (all shorted survival) | | - | | S | | - | 7 |
| Bao et al. [28] | 2014 | China | - | - | - | REMBRANDT, CGGA | - | PDE4C (L) | | - | | L | | - | 6 |
| Xing et al. [29] | 2015 | China | RT-qPCR | 1.1 | 90, NR | - | - | Notch-1, EGFR (L) | | - | | L | | - | 7 |
| Sibin et al. [30] | 2015 | India | RT-qPCR | 1.6 | 21, NR | - | - | CDKN2A (L) | | - | | L | | - | 7 |
| Cai et al. [31] | 2015 | China | - | - | - | CGGA | TCGA | CXCL10, IL17R, CCR2, IL17B, IL10RB, CCL2 (all shorted survival) | | - | | S | | - | 8 |
| Zhou et al. [32] | 2015 | USA | - | - | - | TCGA | - | CD151 (S), α3 integrin (S) | | - | | S | | - | 6 |
| Sibin et al. [33] | 2015 | India | RT-qPCR | 1.4 | 21, NR | - | - | BMI1 (L) | | CD133 (L) | | L | | - | 7 |
| Liu et al. [34] | 2015 | China | - | - | - | TCGA | - | PTK7 (S) | | - | | S | | - | 6 |
| Frei et al. [35] | 2015 | Switzerland | RT-qPCR | 1.1 | 64,58 | TCGA | - | TGF-β2 (S) | | - | | S | | - | 7 |
| Bache et al. [36] | 2015 | China | RT-qPCR | 0.9 | 34,58.5 | - | - | CA9, HIF-2α, GLUT-1, OPN (Gene panel; S) | | - | | S | | - | 7 |
| Delic et al. [37] | 2015 | China | RT-qPCR | NR | 25, NR | - | - | CMTM1, CMTM 3, CMTM 6, CMTM 7 (all shorted survival) | | - | | S | | - | 7 |
| Stegen et al. [38] | 2015 | Germany | - | - | - | TCGA | - | IK (S) | | - | | S | | - | 6 |
| Zhang et al. [39] | 2015 | UK | - | - | - | GSE4271, GSE4412, GSE7696 | - | VEGFA, FLT1, KDR (Gene panel; S) | | - | | S | | - | 6 |
| Cheng et al. [40] | 2016 | China | - | - | - | - | TCGA, CGGA | FOXO3, IL6, IL10, ZBTB16, CCL18, AIMP1, FCGR2B, MMP9 (Gene panel, S) | | | | S | | - | 8 |
| Li et al. [41] | 2016 | China | - | - | - | - | CGGA, GSE16011 | - | | MMP9 (L) | | L | | - | 8 |
| Gao et al. [42] | 2016 | China | RT-qPCR | NR | 32, NR | GSE4412, GSE7696 | GSE4412, TCGA | PPIC, EMP3, CHI3L1 (all shorted survival) | | - | | S | | - | 9 |
| Lin et al. [43] | 2016 | China | - | - | - | CGGA, GSE16011 | - | Vimentin (S) | | - | | S | | - | 6 |
| Wang et al. [44] | 2016 | China | - | - | - | TCGA | CGGA | PD-L1 (S) | | - | | S | | - | 8 |
| Nduom et al. [45] | 2016 | USA | - | - | - | TCGA | - | PD-L1 (S) | | - | | S | | - | 6 |
| Bayin et al. [46] | 2016 | USA | - | - | - | TCGA | - | GPR133 (S) | | - | | S | | - | 6 |
| Cheng et a. [47] | 2016 | China | - | - | - | CGGA | GSE16011 | HSPB11 (S) | | - | | S | | - | 8 |
| Wang et al. [48] | 2016 | China | - | - | - | CGGA | TCGA | FGFR3 (L) | | - | | L | |  | 8 |
| Steponaitis et al. [49] | 2016 | Lithuania | RT-qPCR | 0.78 | 38, NR | - | - | CHI3L1 (S) | | - | | S | | - | 7 |
| Sun et al. [50] | 2016 | China | - | - | - | CGGA | TCGA, EMBRANDT, GSE16011 | KIF23 (S) | | - | | S | | - | 8 |
| Kolodziej et al. [51] | 2016 | Germany | RT-qPCR | 2.4 | 44, 57.4 | - | - | NDRG4 (S) | | NDRG2(S) | | S | | - | 7 |
| Codó et al. [52] | 2016 | Switzerland | - | - | - | - | TCGA | GDF-15 (S) | | - | | S | |  | 8 |
| Wang et al. [53] | 2016 | China | - | - | - | CGGA | TCGA, EMBRANDT, | KCNN4 (S) | | KCNB1 , KCNJ10 (Gene panel, S) | | S | | - | 8 |
| Murnyák et al. [54] | 2017 | Hungary | - | - | - | - | TCGA | PARP1(S) | | - | | S | | - | 8 |
| Xu et al. [55] | 2017 | China | - | - | - | - | TCGA, Rembrandt | CCDC109B (S) | | - | | S | | - | 6 |
| Zhu et al. [56] | 2017 | Netherland | - | - | - | TCGA | - | P2RY12 (L) | | - | | L | | - | 6 |
| Ohtaki et al. [57] | 2017 | Japan | RT-qPCR | NR | 25, NR | - | - | ACTC1 (S) | | - | | S | | - | 7 |
| Zhai et al. [58] | 2017 | USA | - | - | - | REMBRANDT, TCGA | - | IDO1 (S) | | - | | S | | - | 6 |
| Soni et al. [59] | 2017 | India | RT-qPCR | 2.0 | 51, NR | - | - | CD24 , Nanog (all shorted survival) | | - | | S | | - | 7 |
| Haynes et al. [60] | 2017 | UK | - | - | - | TCGA | - | PPARA (L) | | - | | L | |  | 6 |
| Huang et al. [61] | 2017 | Taiwan | - | - | - | TCGA | CGGA | BICD1 (S) | | - | | S | |  | 8 |
| Kim et al. [62] | 2017 | Republic of Korea | - | - | - | TCGA | - | GLI1, PTCH1, SMO (all shorted survival) | | - | | S | |  | 6 |
| Wang et al. [63] | 2017 | USA | - | - | - | TCGA | - | APOBEC3G (S) | | - | | S | | - | 6 |
| Han et al. [64] | 2017 | China | - | - | - | TCGA | CGGA, Rembrandt, Gravendeel [64] | TAGLN2 (S) | | - | | S | |  | 8 |
| Feldheim et al. [65] | 2018 | Germany | RT-qPCR | 1.7 | 51, 59 | - | - | ATF5(S) | | - | | S | | - | 7 |
| Jia et al. [66] | 2018 | China | - | - | - | TCGA | CGGA | C3AR1, C3AR5, CD33, COLA62, ICAM1, IL1R2, IL7R, ITGB2, PCOLCE, TANAIP2, TANAIP6 (all shorted survival) | | - | | S | | - | 8 |
| Yue et al. [67] | 2018 | China | - | - | - | TCGA | - | EMP3 (S) | | - | | S | | - | 6 |
| Wang et al. [68] | 2018 | China | - | - | - | TCGA | CGGA | C9orf64, OSMR,  MDK, MARVELD1, PTRF, MYD88, BIRC3, RPP25 (all shorted survival) | | - | | S | |  | 8 |
| Xu et al. [69] | 2018 | China | - | - | - | TCGA | GSE16011 | OSMR, MED10 , PTPRN (all shorted survival), SOX21 (L) | | - | | S,L | | AUC values =0.905 | 8 |
| Roy et al. [70] | 2018 | Canada | - | - | - | TCGA | - | TGF-β₁ (S) | | - | | S | | - | 6 |
| Chen et al. [71] | 2018 | China | - | - | - | CGGA, GSE53733 | TCGA | HOXB2, ANXA1, HOXB7, WDR63, DSG2, TNNT1 (all shorted survival); PDE8B, FXYD6, SEZ6L, ABCG2, NCKAP5, SLC25A48, FREM3, HES6, BMP2, MSTN, FERMT1 (all longer survival) | | - | | S,L | | - | 8 |
| Shu et al. [72] | 2018 | China | - | - | - | TCGA | CGGA, GSE16011 | WEE1, EMP3, IGF2BP3 (all longer survival) | | - | | L | | - | 8 |
| Liu et al. [73] | 2018 | China | - | - | - | GSE53733 | GSE50161 | LINGO1 (L), C7orf31(S), VEGFA(S) | | - | | S,L | | - | 8 |
| Zhang et al. [74] | 2018 | China | - | - | - | TCGA, GSE22866 | CGGA | PRKCG, PRKCB CAMK2A(all shorted survival) | | - | | S | | - | 8 |
| Gilder et al. [75] | 2018 | USA | - | - | - | TCGA | - | PLAUR (S) | | - | | S | | - | 6 |
| Takashima et al. [76] | 2018 | Japan | - | - | - | TCGA | - | PD-L1 (S) | | - | | S | | - | 6 |
| Han et al. [77] | 2018 | USA | - | - | - | TCGA | - | IL-13Rα1 (S), IL-13Rα2 (S) | | - | | S | |  | 6 |
| Vasaikar et al. [78] | 2018 | Sweden | - | - | - | TCGA, GEO | - | ETBR (S) | | - | | S | |  | 6 |
| Cheng et al. [79] | 2018 | Taiwan. | - | - | - | GEO | - | PSMB4 (S) | | - | | S | |  | 6 |
| Zhang et al. [80] | 2018 | China | - | - | - | TCGA, GEO | - | CDK1, CCNB1 , CDC20 (all shorted survival) | | - | | S | |  | 6 |
| Breznik et al. [81] | 2018 | Slovenia | - | - | - | TCGA | - | cathepsin X (S), cathepsin K (S) | | - | | S | |  | 6 |
| Cho et al. [82] | 2018 | China | RT-qPCR | 1.4 | 60, 54.2 | - | - | CD49d (S) | | - | | S | | - | 7 |
| Gao et al. [83] | 2018 | China | - | - | - | TCGA, CGGA | - | ISG20 (S) | | - | | S | | - | 6 |
| Zhou et al. [84] | 2018 | China | - | - | - | - | GSE15824 , GSE51062 | NLRP2 (S), GPR1 (S), RANBP17 (L), | | - | | L ,S | |  | 8 |
| Guan et al. [85] | 2018 | China | - | - | - | TCGA, CGGA | - | SLC9A1 (S) | | - | | S | | - | 6 |
| Zhang et al. [86] | 2018 | China | - | - | - | CGGA | TCGA | B7-H3 (S) | | - | | S | | - | 8 |
| Zhong et al. [87] | 2018 | China | - | - | - | GSE42656, GSE50161, GSE86574, CGGA | - | AURKA, NDC80, KIF4A, and NUSAP1 (all shorted survival) | | - | | S | |  | 6 |
| Du et al. [88] | 2018 | USA | - | - | - | TCGA | Rembrandt, GSE4271, GSE4412 | FBXO17 (S) | | - | | S | | - | 8 |
| Wang et al. [89] | 2018 | China | Microarray | 2.1 | 102, NR | - | TCGA | GPR65 (S) | | - | | S | | - | 9 |
| Werner et al. [90] | 2019 | Germany | - | - | - | - | TCGA | CD40(S) | | - | | S | | - | 8 |
| Guo et al. [91] | 2019 |  | - | - | - | TCGA | GSE13041 | NMB, RTN1, GPC5, EMP3 | |  | |  | | AUC values =0.867 | 8 |
| Yin et al. [92] | 2019 | China | - | - | - | - | TCGA, GSE7696, GSE13041 | G6PC3 (S), IGFBP2 (S), TIMP4(L) | | PTPRN (L) , RGS14 (L) | | L ,S | | AUC values =0.704 | 8 |
| Cao et al. [93] | 2019 | China | - | - | - | TCGA | GEO (GSE4290, GSE16011, GSE59612, GSE90604) | OSMR, HOXC10, SCARA3 (all shorted survival) | | SLC39A10 (L) | | L ,S | | - | 8 |
| Zuo et al. [94] | 2019 | China | - | - | - | - | TCGA, CGGA | CD79B, MAP2K3, IMPDH1, SLC16A3, MPZL3 , APOBR (all shorted survival) | | - | | S | | AUC values = 0.699 -0.779 | 8 |
| Cheng et al. [95] | 2019 | China | - | - | - | TCGA | GSE74187, CGGA | MEOX2, SNAI2, ZNF22(all shorted survival) | | LHX2 (S), | | L ,S | | AUC = 0.734 for 1-year prediction | 8 |
| Hsu et al. [96] | 2019 | Taiwan | - | - | - | TCGA | GSE16011, GSE4412, GSE4271 | CTSZ, EFEMP2, ITGA5, KDELR2, MDK, MICALL2, MAP 2 K3, PLAUR, SERPINE1, SOCS3(all shorted survival) | | - | | S | | - | 8 |
| Wang et al. [97] | 2019 | China | - | - | - | TCGA | CGGA | - | | ITGA3, NRG1, MAP1LC3A (all longer survival) | | L | | AUC = 0.739 for 1-year prediction | 8 |
| Yuan et al. [98] | 2019 | China | Microarray | 1.6 | 180, NR | - | - | IGFBP2 (S) | | - | | S | | - | 7 |
| Masiulionytė et al. [99] | 2019 | Lithuania | RT-qPCR | 18/20 | 38, NR | - | - | MT1A, MT1X, MT2, MT3(all shorted survival) | | - | | S | | - | 7 |
| Morelli et al. [100] | 2019 | Italy | RT-qPCR | NR | 66, NR | - | - | TRPML1(S) | | - | | S | | - | 7 |
| Kruthika et al. [101] | 2019 | India | - | - | - | - | TCGA, Rembrandt | MYL9 (S) | | - | | S | | - | 8 |
| Liu et al. [102] | 2019 | China | - | - | - | - | TCGA | LAMC1 (S) | | - | | S | | - | 8 |
| Yang et al.[103] | 2019 | China | - | - | - | - | TCGA, GEO | HOXB2, BET1, PXN, TIMP4 (all NR) | | NRXN3 (L), NPTX2(L), CRELD1 (NR), SV2B(NR), MPO(NR), SCN1B(NR) | | L, NR | | - | 8 |
| Liu et al.[104] | 2019 | China | - | - | - | - | TCGA, CGGA | CD163 (S) | | - | | S | | - | 8 |
| Zhou et al.[105] | 2019 | China | - | - | - | TCGA, GSE4290, GSE 50161 | - | RRM2 (S) | | - | | S | | - | 6 |
| Wang et al.[106] | 2019 | USA | - | - | - | TCGA | - | NT5E(S) | | - | | S | | - | 6 |
| Fu et al. [107] | 2019 | China | - | - | - | TCGA | - | - | | PHYHIPL (S) | | S | | - | 6 |
| Hasan et al. [108] | 2019 | USA | - | - | - | TCGA, IGAP | - | IL-8 (S) | | - | | S | | - | 6 |
| Ye et al. [109] | 2019 | China | RT-qPCR | NR | 178, NR | TCGA, GEO, CGGA | 178 Xiangya GBM patients | PCNA, CALCOCO2, ADI1, GPNMB, MTDH, EIF3D, NEDD9, ERCC1, PLOD1, ATP6V1E1, VTI1B, ECHS1, RCAN2 (Gene panel; S) | | | | S | | AUC = 0.696 for TCGA | 8 |
| Zeng et al. [110] | 2019 | China | - | - | - | CGGA | - | RELB (S) | | - | | S | | - | 6 |
| Zhang et al. [111] | 2019 | China | RT-qPCR | NR | 22, NR | CGGA, GSE16011 | TCGA | PLK4 (S) | | - | | S | | - | 9 |
| Chen et al. [112] | 2019 | China | - | - | - | TCGA, GTEx | - | CTRP1(S) | | - | | S | | - | 6 |
| Tang et al. [113] | 2019 | China | - | - | - | TCGA | - | FOXM1 (S), Survivin (S) | | - | | S | | - | 6 |
| Waterset al. [114] | 2019 | USA | - | - | - | TCGA | - | IL-1 (S), OSM (S) | | - | | S | | - | 6 |
| Yuan et al. [115] | 2019 | China | - | - | - | TCGA, GDS3885, French dataset | - | Orai2 (S) | | - | | S | | - | 6 |
| Yang et al. [116] | 2019 | China | - | - | - | ONCOMINE and Oncolnc databases | - | HOXC6 (S) | | - | | S | | - | 6 |
| Zeng et al. [117] | 2019 | China | - | - | - | TCGA, GSE4412 | - | UGP2 (S) | | - | | S | | - | 6 |
| Xu et al. [118] | 2019 | China | - | - | - | TCGA, GSE16011 | - | TMEM168 (S) | | - | | S | | - | 6 |
| Fernández-García et al. [119] | 2019 | Spain | - | - | - | TCGA, REMBRANDT | - | SMS1 (S), SMS2(S) | | - | | S | | - | 6 |
| Luo et al. [120] | 2019 | China | - | - | - | GSE13276, GSE116520 | GEPIA | VEGFA (S), CXCL8 (S) | | - | | S | |  | 8 |
| Ji et al. [121] | 2019 | China | - | - | - | TCGA | - | GBP1 (S) | | - | | S | | - | 6 |
| Dong et al. [122] | 2019 | China | - | - | - | GEPIA | - | MLKL (S) | | - | | S | | - | 6 |
| Wang et al. [123] | 2019 | China | RT-qPCR | 0.57 | 33, 44 | - | - | MX2 (L) | | - | | L | | - | 7 |
| Wang et al. [124] | 2019 | China | - | - | - | TCGA | - | LGALS3 (S) | | - | | S | | - | 6 |
| Steponaitis et al. [125] | 2019 | Lithuania | RT-qPCR | 0.65 | 102, 61.7 | - | - | AREG (S) | | - | | S | | - | 7 |
| Wang et al. [126] | 2019 | China | - | - | - | TCGA, CGGA | - | TMEM71 (S) | | - | | S | | - | 6 |
| Zhang et al. [127] | 2019 | China | - | - | - | TCGA | - | LKB1 (L) | | - | | L | | - | 6 |
| Jovčevska et al. [128] | 2019 | Slovenia | - | - | - | TCGA | - | FREM2 (L) | | - | | L | | - | 6 |
| Komaki et al. [129] | 2019 | Japan | RT-qPCR | 1.9 | 52, 65.9 | - | - | GLUT1 (S) | | - | | S | | - | 7 |
| Sharma et al. [130] | 2019 | India | - | - | - | TCGA | - | NLRP12 (S) | | - | | S | | - | 6 |
| Prasad et al. [131] | 2020 | UK | - | - | - | - | GEO (GSE4290, GSE12657, GSE13276, GSE19728, GSE90886, GSE108474, GSE116520), TCGA, ArrayExpress | IGFBP2 (S) | | SLC39A10 (L), PTPRN(S), STEAP2 (S) | | S, L | | AUC = 0.766 for 1-year prediction | 8 |
| Vachher et al. [132] | 2020 | India | - | - | - | - | TCGA | RETN (S), GRN (S) | | - | | S | | - | 8 |
| Nesterova et al. [133] | 2020 | USA | - | - | - | - | TCGA | HFE (S) | | - | | S | | - | 8 |
| Haddad et al. [134] | 2020 | USA | - | - | - | - | TCGA | CYT score (GZMA and PRF1) (S) | | - | | S | | - | 8 |
| Sun et al. [135] | 2020 | China | - | - | - | - | TCGA | - | | ABCC3 (S) | | S | | - | 8 |
| Du et al. [136] | 2020 | China | RT-qPCR | NR | 28, NR | - | TCGA, CGGA, CPTAC | FAM20C(S) | | - | | S | | - | 9 |
| Pan et al. [137] | 2020 | China | - | - | - | GSE16011, TCGA | CGGA | GRIA2(S), RYR3(S) | | - | | S | | AUC = 0.675 for 2-year prediction | 8 |
| Valiulyte et al. [138] | 2020 | Lithuania | RT-qPCR | NR | 59, NR | - | TCGA | SEMA3A, SEMA3F, ITGB3, ITGA5, VEGFA (all shorter survival) and SEMA3D , SEMA3G (all longer survival) | | - | | S, L | | AUC = 0.810 for 1-year prediction | 9 |
| Liu et al. [139] | 2020 | China | - | - | - | CGGA | TCGA | PDCD1 (S) | | - | | S | | - | 8 |
| Zhu et al. [140] | 2020 | China | - | - | - | TCGA | - | C1s ,HSD3B7 (all shorter survival) | | - | | S | | - | 6 |
| Faried et al. [141] | 2020 | China | - | - | - | TCGA | CGGA, REMBRANDT | POSTN (S) | | - | | S | | - | 8 |
| Hu et al. [142] | 2020 | China | - | - | - | TCGA | - | AR (S) | | - | | S | | - | 6 |
| Li et al. [143] | 2020 | China | - | - | - | TCGA, GTEx | GEPIA | WDR12 (S) | | - | | S | | - | 8 |
| Yang et al. [144] | 2020 | China | - | - | - | TCGA, GSE12657, GSE15824, GSE42656, GSE50161 | - | SLC12A5, CCL2, IGFBP2, PDPN (all shorter survival) | | - | | S | | AUC = 0.701 | 6 |
| Yarmishyn et al. [145] | 2020 | Taiwan | - | - | - | Oncomine, TCGA | - | MSI1, YTHDF1 (all shorter survival) | | - | | S | |  | 6 |
| Gan et al. [146] | 2020 | China | RT-qPCR | NR | 30, NR | TCGA | - | ALDH1A3 (S) | | - | | S | | - | 7 |
| Peng et al. [147] | 2020 | China | - | - | - | TCGA, GTEx | - | LYZ , FPR3 , FBP1 , GPSM3 , CCR1 , HAVCR2 , MNDA , MSR1 , PIK3AP1 , LCP1 , C-3AR1 SAMSN1 , BCL2A1 (Gene panel) | | - | | S | | AUC = 0.751 | 6 |
| Song et al. [148] | 2020 | China | - | - | - | TCGA, CGGA | - | CTGF (S) | | - | | S | | - | 6 |
| Yang et al. [149] | 2020 | China | - | - | - | mRNAseq_693 dataset | - | CCNB1, CDC20, BUB1, CCNA2 (all shorter survival) | | - | | S | |  | 6 |
| Wu et al. [150] | 2020 | China | - | - | - | TCGA, CGGA | - | DGCR5 (L) | | - | | L | | - | 6 |
| Shi et al. [151] | 2020 | China | - | - | - | GEO | - | BST2 (S) | | - | | S | | - | 6 |
| Mao et al. [152] | 2020 | China | - | - | - | TCGA, CGGA, GSE16011 | - | KDELR2 (S) | | - | | S | | - | 6 |
| Fuentes-Fayos ET AL. [153] | 2021 | Spain | RT-qPCR | NR | 29, NR | - | CGGA, Murat dataset | SRSF3 (S) | | - | | S | | - | 9 |
| Miao et al. [154] | 2020 | China | - | - | - | TCGA, GEO | - | PDCD1 (S) | | - | | S | | - | 6 |
| Chi et al. [155] | 2020 | China | - | - | - | GSE43378, GEPIA | - | WBSCR22 (S) | | - | | S | | - | 6 |
| Kao et al. [156] | 2021 | Taiwan | RT-qPCR | NR | NR | - | TCGA, CGGA, GEO | SGO2 (S) | | - | | S | | - | 9 |
| Yi et al. [157] | 2021 | China | - | - | - | - | TCGA, CGGA, GSE16011, GSE4290, GSE108474 | BCAT1 (S) | | - | | S | | - | 8 |
| Geraldo et al. [158] | 2021 | France | - | - | - | - | TCGA | SLIT2 (S) | | - | | S | |  | 8 |
| Fang et al. [159] | 2021 | USA | - | - | - | - | TCGA, REMBRANDT, French glioma | YTHDF2 (S) | | - | | S | |  | 8 |
| Jiang et al. [160] | 2021 | China | - | - | - | TCGA | CGGA | BCL3, C2orf29, CD163, F3, FCGR2B, HRH1, PRAM1, SLC16A3, SOCS3, TREM1 (all shorter survival) | | - | | S | | - | 8 |
| Wang et al. [161] | 2021 | China | - | - | - | TCGA, CGGA, GSE4412, GSE43378 | GSE13041 | - | | CDHR1 (S) | | S | | - | 8 |
| Maimaiti et al. [162] | 2021 | China | - | - | - | TCGA, CGGA, | - | - | | DLL3 (S) | | S | | - | 6 |
| Wang et al. [163] | 2021 | China | - | - | - | TCGA, ONCOMINE | - | FAM46A (S) | | - | | S | | - | 6 |
| Zhao et al. [164] | 2021 | China | - | - | - | - | TCGA, GTEx | CDC6(S) | | - | | S | | AUC = 0.584 for 1-year prediction | 8 |
| Morrison et al. [165] | 2021 | USA | - | - | - | - | GEO | RAD51 (S) | | - | | - | | - | 8 |
| Xu et al. [166] | 2021 | China | - | - | - | TCGA | GEO, CGGA | FN1 (S) , CXCL8 (S) | | - | | S | | - | 8 |
| Jeong et al. [167] | 2021 | Republic of Korea | - | - | - | - | TCGA, GEO, IGAP | RFX4 (S) | | - | | S | | - | 8 |
| Cao et al. [168] | 2021 | China | RT-qPCR | NR | 75, NR | TCGA, GEO | CGGA | GRPR, CXCL5, CXCL11 (all longer survival) | | - | | S | | - | 9 |
| Cheng et al. [169] | 2021 | China | - | - | - | TCGA, GEO | CGGA | PRDX1 (S), SEC61B (S), XRCC5(S) | | BCL2L2 (S) | | S | | - | 8 |
| Hu et al. [170] | 2021 | China | - | - | - | TCGA, CGGA | - | SLC7A11 (L) | | - | | L | |  | 6 |
| Phan et al. [171] | 2021 | Republic of Korea | - | - | - | Gravendeel dataset, GSE4536 | - | RGMA (S) | | - | | S | | - | 6 |
| Hu et al. [172] | 2021 | China | - | - | - | TCGA | TCGA internal validation set,  GSE74187, GSE4412 | CTSB, NFKBIZ,  TNFSF14, CXCL2, SEMA4F, OSMR (all longer survival), BMPR1A (L) | | - | | S,L | | AUC = 0.64 (GSE74187) for 1-year prediction | 8 |
| Lan et al. [173] | 2021 | China | - | - | - | TCGA | GSE16011, GSE13041 | TCF3 (L), IGFBP2, TYRO3, NOD2 (all shorter survival) | | - | | S, L | |  | 8 |
| Łysiak et al. [174] | 2021 | Sweden | - | - | - | TCGA | - | SRY (S) | | - | | S | | - | 6 |
| Chen et al. [175] | 2021 | Taiwan | - | - | - | TCGA, CGGA | - | TRIP13 (S) | | - | | S | | - | 6 |
| Hsieh et al. [176] | 2021 | Taiwan | - | - | - | GSE4290, GSE13041 | - | CTSD (S), LCN2 (L) | | - | | S, L | | - | 6 |
| Shi et al. [177] | 2021 | China | - | - | - | TCGA, GEO, CGGA | - | EFNB1 (S) | | - | | S | | - | 6 |
| Yang et al. [178] | 2021 | China | - | - | - | TCGA, GSE90598, | - | GABRD (S), SYT1 (S) | | - | | S | | - | 6 |
| Zhang et al. [179] | 2021 | China | - | - | - | CGGA, GSE43378 | - | TNFRSF12A (S) | | - | | S | | - | 6 |
| Urbantat et al. [180] | 2021 | Germany | - | - | - | TCGA, | - | VEGF, CXCL2, IL8 (all longer survival) | | - | | S | | - | 6 |
| Krassnig et al. [181] | 2021 | Austria | - | - | - | TCGA | - | eIF4H (S) | | - | | S | | - | 6 |
| Flor et al. [182] | 2021 | USA | - | - | - | TCGA | - | CAT (S) | | - | | S | | - | 6 |
| Cocola et al. [183] | 2021 | Italy | - | - | - | TCGA | - | - | | TMEM230 (S) | | S | | - | 6 |
| Cheng et al. [184] | 2021 | China | - | - | - | CGGA, TCGA, GTE | - | DCBLD2 (S) | | - | | S | | - | 6 |
| Liu et al. [185] | 2021 | China | - | - | - | - | CGGA, TCGA | BCL7A (S) | | - | | S | | - | 8 |
| Li et al. [186] | 2021 | China | - | - | - | TCGA | GSE13041, GSE83300, | ATF7IP (L), CCNB1IP1(L), LBH (S) | | - | | S,L | | AUC = 0.71 (GSE13041) for 1-year prediction | 8 |
| Ye et al. [187] | 2021 | China | - | - | - | - | TCGA | AP1S3 (S) | | - | | S | | - | 8 |
| Zhou et al. [188] | 2021 | China | - | - | - | CGGA | - | FXYD2 (L) | | - | | L | | - | 6 |
| Tu et al. [189] | 2021 | China | - | - | - | TCGA | CGGA, GSE16011 | ANG, APOBEC3F, CARHSP1, FBXO17, ISG20, KHNYN, LSM12, PABPC3, PNRC2, RDM1, SPATS2L, TTF2, CTIF, GNL1 (Gene panel; S) | | | | S | | AUC = 0.871 (TCGA) for 1-year prediction | 8 |
| Zhou et al. [190] | 2021 | China | - | - | - | TCGA, CGGA | - | LMNB1, DLGAP5 (all shorter survival) | | - | | S | | - | 6 |
| Moriconi et al. [191] | 2021 | UK | - | - | - | TCGA, CGGA | - | CAV1 (S) | | - | | S | | - | 6 |
| Stanke et al. [192] | 2021 | USA | - | - | - | REMBRANDT | - | HK2 (S), PKM2(S), G6PD(L), PGLS(L), SDHB(L), COX5A (L) | | - | | S,L | | - | 6 |
| Redekar et al. [193] | 2022 | China | - | - | - | GSE111260, GSE103227, GSE104267 | TCGA, HPA | CALN1, ELAVL3, ADCY3, SYN2, ARL13B, SLC12A5, SOD1 (all longer survival) | | CETN2, MKI67, ARL13B, SETDB1 (all longer survival) | | L | | - | 8 |
| Liao et al. [194] | 2022 | China | - | - | - | - | TCGA, GTEx | PRMT3 (S) | | - | | S | | - | 8 |
| Zhao et al. [195] | 2022 | China | - | - | - | - | TCGA, CGGA, GSE 162631 | TUBA1C (S), KDELR2 (S) | | RPS4X (S), SLC40A1(S) | | S | | AUC = 0.655 (TCGA) and 0.587 (CGGA) for 1-year prediction | 8 |
| Nimbalkar et al. [196] | 2022 | India | RT-qPCR | - | 34, NR | - | TCGA, REMBRANDT | CHI3L2 (S) | | - | | S | | - | 9 |
| Wu et al. [197] | 2022 | China | - | - | - | TCGA, GSE182109 | CGGA | CTSD, AP1S1, YWHAG, IER3 (all shorter survival) | | - | | S | | AUC = 0.76 for 1-year prediction | 8 |
| Zhang et al. [198] | 2022 | China | - | - | - | GSE50161, GSE116520 | - | LOX, SERPINH1 , TGFBI (all shorter survival) | | - | | S | | - | 6 |
| Pan et al. [199] | 2022 | China | - | - | - | - | ONCOMINE | SOX3(L) | | - | | L | | - | 8 |
| Y uan et al. [200] | 2022 | China | - | - | - | CGGA | TCGA | BCAT1, HPX, NNMT, TBX5, RAB42,  TNFRSF19(all shorter survival) | | C16orf86 ,  TRPC5 (all longer survival) | | S,L | | AUC = 0.712 (TCGA) and 0.635 (CGGA) for 1-year prediction | 8 |
| Jin et al. [201] | 2022 | China | - | - | - | CGGA693 | CGGA325, TCGA | MSH2 (S), CNTRL(S) | | - | | S | | - | 8 |
| Li et al. [202] | 2022 | China | - | - | - | TCGA | TCGA, GSE16011 | HOXC6(S) | | - | | - | | - | 8 |
| Santoni et al. [203] | 2022 | Italy | RT-qPCR | 0.9 | 66, NR | - | - | TRPML2(S), TRPML1(S) | | - | | S | | - | 7 |
| Dubois et al. [204] | 2022 | Netherland | - | - | - | - | TCGA | STAT5b(S) | | - | | S | | - | 8 |
| Trivedi et al. [205] | 2022 | India | RT-qPCR | 1.75 | 55, NR | - | - | STAT3 (L) | | - | | L | | - | 7 |
| Xiong et al. [206] | 2022 | China | - | - | - | - | TCGA, REMBRANDT | ETV6 (S) | | - | | S | |  | 8 |
| Zhang et al. [207] | 2022 | China | - | - | - | TCGA, GEO | - | DARS (L), GDI2 (L), P4HA2 (S), TRUB1(L) | | - | | S,L | | - | 6 |
| Wang et al. [208] | 2022 | China | - | - | - | TCGA, CGGA | - | MMP9(S), SLC16A3 (S) | | - | | S | | - | 6 |
| Park et al. [209] | 2022 | Republic of Korea | - | - | - | REMBRANDT, IGAP | - | SPRY1 (S) | | - | | S | | - | 6 |
| Moreno et al. [210] | 2022 | Brazil | - | - | - | TCGA | - | SAA1, TNFSF14, F13A1, IFITM1, S100A8, CLEC5A, C3, SERPING1, C2, THBD, CCR1, LY96, IL32, CD14, TNFSF13B, SLC11A1, CTSL, CD4, CD68, ENG, ICOSLG, TAPBP (all shorter survival) | | - | | S | |  | 6 |
| Akçay et al. [211] | 2022 | Turkey | - | - | - | TCGA, GSE124145 | - | GUCA1A, RFC2, GNG11, MMP19, NRG1 (all shorter survival) | | - | |  | |  | 6 |
| Liu et al. [212] | 2022 | China | - | - | - | TCGA, GTEx | - | - | | ABLIM1 (S) | | S | | - | 6 |
| Lin et al. [213] | 2022 | China | - | - | - | GSE43289 | - | TNFAIP6 (S) | | - | | S | |  | 6 |
| Güven et al. [214] | 2022 | Turkey | - | - | - | TCGA | GSE124145 | IL1R1, SORBS2, S100A8, CCL8, DAB2 (all shorter survival) | | - | | S | | - | 8 |
| Zheng et al. [215] | 2022 | China | - | - | - | TCGA, CGGA | - | CBX8 (S) | | CBX7 (L) | | S | | - | 6 |
| Liu et al. [216] | 2022 | China | RT-qPCR | NR | 17, NR | TCGA | - | IL17RD (L) | | - | | L | | - | 7 |
| Lin et al. [217] | 2022 | China | - | - | - | TCGA, CGGA | - | CDCP1 (S) | | - | | S | | - | 6 |
| Cui et al. [218] | 2022 | China | - | - | - | GEO | CGGA | SAA1 (S) | | - | | S | | - | 8 |
| Gao et al. [219] | 2022 | China | - | - | - | TCGA, GTEx | CGGA | CCL2, CCL8, CCL18, CCL28, CXCL1, CXCL5, CXCL13(all shorter survival) | | - | | S | | - | 8 |
| Han et al. [220] | 2022 | China | - | - | - | TCGA, | - | MAFF, SLC2A3,  HSP90B1, TNFRSF1A, PAK1, DDB2, MDM2, DKK3 (all shorter survival); CTBP2, E2F2, ECSIT, ID4 (all longer survival) | | - | | S | | - | 6 |
| Wei et al. [221] | 2022 | China | - | - | - | TCGA, CGGA | - | TMEM59L (L) | | - | | L | |  | 6 |
| Vedunova et al. [222] | 2022 | Russia | - | - | - | TCGA | CGGA | CFH, GALNT3, SMC4, VAV3 (all longer survival) | | - | | S | | AUC = 0.88 for 1-year prediction | 8 |
| Zhang et al. [223] | 2022 | China | - | - | - | TCGA, CGGA | - | CDK4, S100A16 , TNFRSF10B, BIRC5, ADCYAP1R1, BMP1, TNFRSF19, APOBEC3C, (all shorter survival); ANGPTL2(L), BMP2(L), | | MET (S), SSTR2 (S), VGF (S), NRG3(L), JAG2(L) | | S,L | | - | 6 |
| Zhu et al. [224] | 2022 | China | - | - | - | TCGA, CGGA, GEO | - | COL6A2 (S) | | - | | S | | - | 6 |
| Chen et al. [225] | 2022 | China | - | - | - | TCGA, CGGA, GSE16011, GSE74187, GSE82009 | - | MLLT11 (L) | | - | | L | | - | 6 |
| Xie et al. [226] | 2022 | China | - | - | - | TCGA, GEPIA | - | CNN3 (S) | | - | | S | | - | 6 |
| Fuentes-Fayos et al. [227] | 2022 | Spain | - | - | - | CGGA, Rembrandt | - | SF3B1 (S) | | - | | S | | - | 6 |
| Guda et al. [228] | 2022 | USA | - | - | - | TCGA, REMBRANDT | - | Gal-1 (S) | | - | | S | | - | 6 |
| Du et al. [229] | 2022 | China | - | - | - | CGGA, TCGA | - | CD44 (S) | | - | | S | | - | 6 |
| Phillips et al. [230] | 2022 | Germany | - | - | - | TCGA | - | FBXO28 (L) | | - | | L | | - | 6 |
| Li et al. [231] | 2022 | China | - | - | - | GEPIA, TCGA | - | - | | CNPY4 (L) | | L | | - | 6 |
| Li et al. [232] | 2022 | China | - | - | - | CGGA, | - | HMGB1 (S) | | - | | S | | - | 6 |
| Guan et al. [233] | 2022 | China | - | - | - | TCGA | GEPIA2 | TGR5 (S) | | - | | S | | - | 8 |
| Guo et al. [234] | 2022 | China | - | - | - | TCGA | CGGA | TIMM44 (S) | | - | | S | | - | 8 |
| Wang et al. [235] | 2022 | China | - | - | - | CGGA | - | NONO (S) | | - | | S | | - | 6 |
| Wan et al. [236 | 2023 | China | - | - | - | TCGA, GSE84465 | CGGA | ANXA1, COL6A1, PDPN (all shorter survival) | | - | | S | | - | 8 |
| Gao et al. [237] | 2023 | China | - | - | - | TCGA, GSE16011, GSE90604 | - | RBM8A (L) | | - | | L | | - | 6 |
| Liu et al. [238] | 2023 | China | - | - | - | TCGA, | CGGA, GEO | CPQ (S) | | - | | S | | - | 8 |
| Chen et al. [239] | 2023 | China | RT-qPCR | NA | NA | TCGA, GSE116520, GSE4290, GSE68848 | - | SLC12A5 (S) | | - | | S | | - | 7 |
| Zheng et al. [240] | 2023 | China | - | - | - | TCGA, CGGA | - | HOXA1 , HOXA2, HOXA3, HOXA4, HOXA5, HOXA10 , HOXA11 (all shorter survival) | | - | | S | | - | 6 |
| Yoon et al. [241] | 2023 | Republic of Korea | - | - | - | TCGA, | - | AEBP1, C13orf18, C1RL, CBR1, CCL2, CHI3L1, CHL1, CHST2, CLEC5A, DYNLT3, EFEMP2, EMP3, F3, FBXO17, FLJ11286, MSN, NSUN5, PDPN, PGCP, PPCS, SERPING1, SLC25A20, SLC2A10, STEAP3, SWAP70, TIMP1, TMEM22, TRIP6. (all shorter survival); ADAM22, ATP5C1, DHRS2, RAC3, SHANK1 (all longer survival)- | | | | S, L | | - | 6 |
| Danget al. [242] | 2023 | Taiwan | - | - | - | TCGA, | CGGA, GSE43378 | CRNDE, NRXN3, POPDC3, PTPRN, PTPRN2, SLC46A2, TIMP1, TNFSF9 (all shorter survival) | | - | | S | | AUC = 0.76 for 1-year prediction | 8 |
| Singh et al. [243] | 2023 | USA | - | - | - | TCGA | - | TRIB1 (S) | | - | | S | | - | 6 |
| Li et al. [244] | 2023 | China | - | - | - | TCGA | GSE4412, GSE4271 | NMB (L) | | - | | L | | - | 8 |
| Ali et al. [245] | 2023 | USA | - | - | - | TCGA | - | MFRN1 (S) | | - | | S | | - | 6 |
| Jia et al. [246] | 2023 | China | - | - | - | TCGA, GEO | - | FZD1(S) , KLF10 (S) | | - | | S | | - | 6 |
| Ren et al. [247] | 2023 | China | - | - | - | TCGA | - | FAM110C (S) | | - | | S | | - | 6 |
| Zheng et al. [248] | 2023 | China | - | - | - | GEO, TCGA | - | TSPAN4 (S) | | - | | S | | - | 6 |
| Ma et al. [249] | 2023 | China | - | - | - | CGGA, TCGA | GEPIA | TREM-1 (S) | | - | | S | | - | 8 |
| Gao et al. [250] | 2023 | China | - | - | - | TCGA, CGGA | - | TMEM2 (S) | | - | | S | |  | 6 |
| Chen et al. [251] | 2023 | China | - | - | - | TCGA, | CGGA, GEO | NCF2, MTHFS, DUSP6, G6PC3, HOXB2, EN2, LBH, (all shorter survival) | | - | | S | | AUC = 0.816 for 1-year prediction | 8 |
| Ye et al. [252] | 2023 | China | - | - | - | Oncomine, GEPIA, TCGA | - | BRD4 (S) | | - | | S | | - | 6 |
| Zhu et al. [253] | 2023 | China | - | - | - | TCGA | CGGA | PTPRN, OSMR, MYD88, EFEMP2 (gene panel; S) | | - | | S | | AUC = 0.782 (TCGA) for 1-year prediction | 8 |
| Li et al. [254] | 2023 | China | - | - | - | TCGA | - | NKD1 (L) | | - | | L | | - | 6 |
| Lin et al. [255] | 2023 | China | - | - | - | TCGA, GEPIA, TIMER | - | NFE2L2(S), NOX4 (S) | | - | | S | | - | 6 |
| Hu et al. [256] | 2023 | China | - | - | - | CGGA, GEPIA | - | TXLNA (S) | | - | | S | | - | 6 |
| Ding et al. [257] | 2023 | China | - | - | - | TCGA | - | KLHDC7B (S) | | - | | S | | - | 6 |
| Le et al. [258] | 2023 | Japan | - | - | - | TCGA | - | NAA10 (S) | | - | | S | | - | 6 |
| Wang et al. [259] | 2023 | China | - | - | - | TCGA, GTEx | - | ANG (S) | | - | | S | | - | 6 |
| Wu et al. [260] | 2023 | China | - | - | - | TCGA | CGGA301, CGGA325 | RELB (S) | | - | | S | | - | 8 |
| Zhu et al. [261] | 2023 | China | - | - | - | TCGA, GTEx, | - | HSPA5 (S) | | - | | S | | - | 6 |
| Azimi et al. [262] | 2023 | Iran | RT-qPCR | 1.6 | 29, 47.6 | TCGA, CPTAC, GSE147352 | CGGA | SAA1, PITX1, FCGR2C, B3GNT7, CSTA (all shorter survival) | | - | | S | | - | 9 |

NA, not reported; AUC, The area under the ROC Curves (AUC); NOS, Newcastle-Ottawa Scale; RT-qPCR, Reverse transcription-quantitative polymerase chain reaction; OS, Overall survival (S: shorter; L: longer); TCGA, The cancer genome atlas; GEO, Gene Expression Omnibus dataset; GSE, GEO Series ;CGGA, Chinese Glioma Genome Atlas; GTEx, Genotype-Tissue Expression (https://commonfund.nih.gov/GTEx/); ONCOMINE database (www.oncomine.org); OncoLnc (www.oncolnc.org);REMBRANDT (<https://gdoc.georgetown.edu/gdoc>) dataset; French glioma (<https://hgserver1.amc.nl/cgi-bin/r2/main.cgi>) ;HPA, Human Protein Atlas HPA; GTEx, the human Genotype-Tissue Expression (GTEx) database; CPTAC, The Clinical proteomic tumor analysis consortium protein expression profile ( <https://proteo>mics.cancer.gov);IGAP, Ivy Glioblastoma Atlas Project.
